# Understanding microbial community dynamics to improve optimal microbiome selection

**DOI:** 10.1186/s40168-019-0702-x

**Published:** 2019-06-03

**Authors:** Robyn J. Wright, Matthew I. Gibson, Joseph A. Christie-Oleza

**Affiliations:** 1 School of Life Sciences, University of Warwick, Coventry, UK; 2 Department of Chemistry, University of Warwick, Coventry, UK; 3 Medical School, University of Warwick, Coventry, UK

**Keywords:** Artificial microbiome selection, Microbial communities, Microbial ecology, Polymer degradation, Chitin degradation, Ecological succession, Microbial community dynamics

## Abstract

**Background:**

Artificial selection of microbial communities that perform better at a desired process has seduced scientists for over a decade, but the method has not been systematically optimised nor the mechanisms behind its success, or failure, determined. Microbial communities are highly dynamic and, hence, go through distinct and rapid stages of community succession, but the consequent effect this may have on artificially selected communities is unknown.

**Results:**

Using chitin as a case study, we successfully selected for microbial communities with enhanced chitinase activities but found that continuous optimisation of incubation times between selective transfers was of utmost importance. The analysis of the community composition over the entire selection process revealed fundamental aspects in microbial ecology: when incubation times between transfers were optimal, the system was dominated by *Gammaproteobacteria* (i.e. main bearers of chitinase enzymes and drivers of chitin degradation), before being succeeded by cheating, cross-feeding and grazing organisms.

**Conclusions:**

The selection of microbiomes to enhance a desired process is widely used, though the success of artificially selecting microbial communities appears to require optimal incubation times in order to avoid the loss of the desired trait as a consequence of an inevitable community succession. A comprehensive understanding of microbial community dynamics will improve the success of future community selection studies.

**Electronic supplementary material:**

The online version of this article (10.1186/s40168-019-0702-x) contains supplementary material, which is available to authorized users.

## Background

Evolution is able to act upon multiple levels of biological organisation [1-5]. It had previously been contested that a whole microbial community may be used as a unit of selection artificial microbiome selection and that a community may become progressively better at a selective process over successive transfers [2, 6-8]. The artificial selection of a measurable and desirable trait is thought to outperform traditional enrichment experiments, as it bypasses community bottlenecks and reduces stochasticity [8]. Artificial selection has been shown to induce statistically significant responses, in both microcosm [2, 8] and computational ecosystems [3]. While microbial communities with desirable phenotypes have been achieved, the results of these experiments have been limited and the composition of these communities has not generally been determined. The underlying mechanisms and key players in this selection have therefore not been identified, nor have the growth parameters involved (e.g. incubation time) been systematically optimised [9].

Microbial communities are known to go through distinct stages of community succession, where they may see large enrichments of different groups of organisms [10-16]. During the colonisation and degradation of the abundant marine polymer, chitin, three phases of community succession were previously observed: (1) selection of colonising organisms, (2) selection of chitin degraders and (3) chitin degraders are overtaken by cheaters [10]. Cheaters, often called cross-feeders, are organisms that are not metabolically capable of carrying out a particular process themselves, but are able to benefit from the public goods generated by others [17-19]. For example, in the environment, it has been shown that the number of organisms capable of taking up the by-products of chitin hydrolysis is far higher than the number that encodes for chitinases [17]. Furthermore, marine microorganisms ‘leak’ a large diversity of organic matter which in turn may be used by the rest of the community [19, 20] enhancing microbial interdependencies [21]. It has however been previously observed that if the abundance of cheaters becomes sufficiently high, then access to the resource may even be blocked completely [22], leading to a loss of community function.

The undulations between cooperation and competition drive niche-specialisation and higher-level community organisation [23]. The structure of the microbial community has been suggested to significantly alter the observed phenotype in artificial microbiome selection experiments [9]. Community structure may be altered through changes in species' composition or interactions between organisms, ultimately leading to changes in the community phenotype. Computational models have shown that community structure, with [24] or without genetic changes [9], can be responsible for differences between the phenotypes of a community subjected to a directed selection and one that is randomly selected. Hence, the understanding of microbial community ecology suggests that controlling microbial community dynamics is important for achieving a high-functioning microbial community.

In the present study, we aimed to determine the mechanisms behind artificial microbiome selection and early microbial community succession in order to optimise the selection of a process, i.e. chitin degradation. Chitin is one of the most abundant polymers on Earth (i.e. the most abundant polymer in marine ecosystems) constituting a key component in oceanic carbon and nitrogen cycles [25]. Many microorganisms are already known to degrade chitin, and the enzymes and pathways used to do so are well characterised [26]. We found that a microbiome could be artificially evolved to achieve higher chitinase activities, but there were certain methodological caveats to this selection process. We found that the incubation time between transfers needed to be continuously optimised in order to avoid community drift and decay. Microbial community composition was evaluated, and we confirmed that, if transfer times are not continuously optimised, efficient biodegrading communities are rapidly taken over by cheaters and predators with a subsequent loss of degrading activity.

## Results

**Fig. 1:**
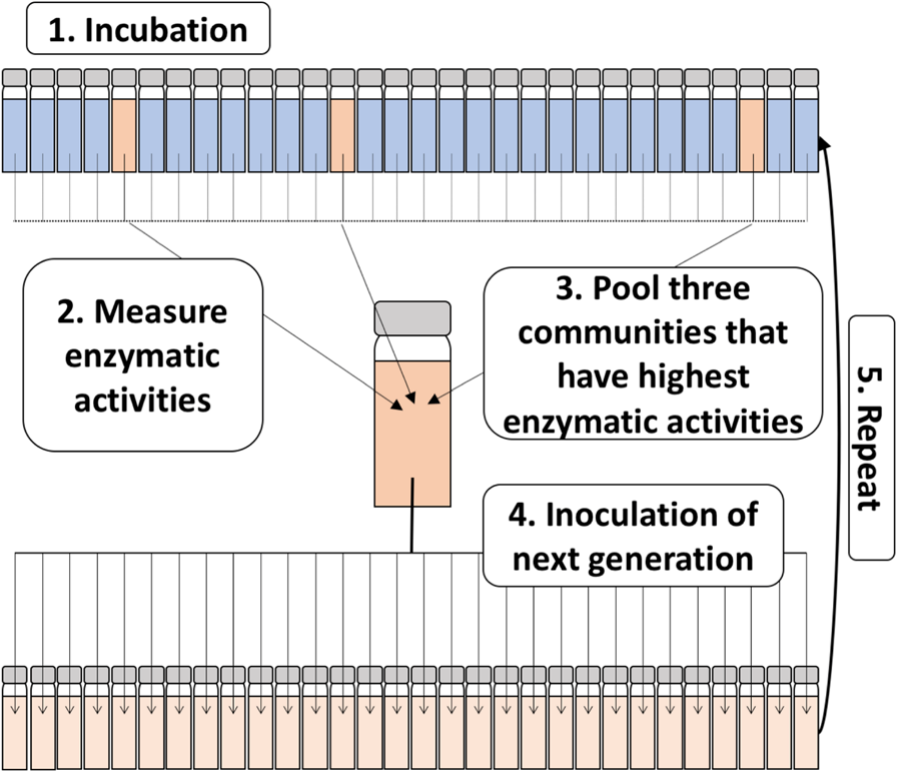
Method used for artificial selection of microbial communities. Briefly, 30 microcosms are inoculated with a natural community found in seawater (1). At the end of the incubation period, the enzymatic activity for a desired trait (e.g. chitinase activity) is measured for each microcosm (2). The three microcosms with the highest enzymatic activities are selected and pooled (3) and used to inoculate the next generation (4). This process is repeated over *n* generations (5)

The experimental setup for artificially selecting microbial communities is depicted in Fig. 1 (see the Materials and methods section for more details).

### First artificial selection experiment: process optimisation

**Fig. 2:**
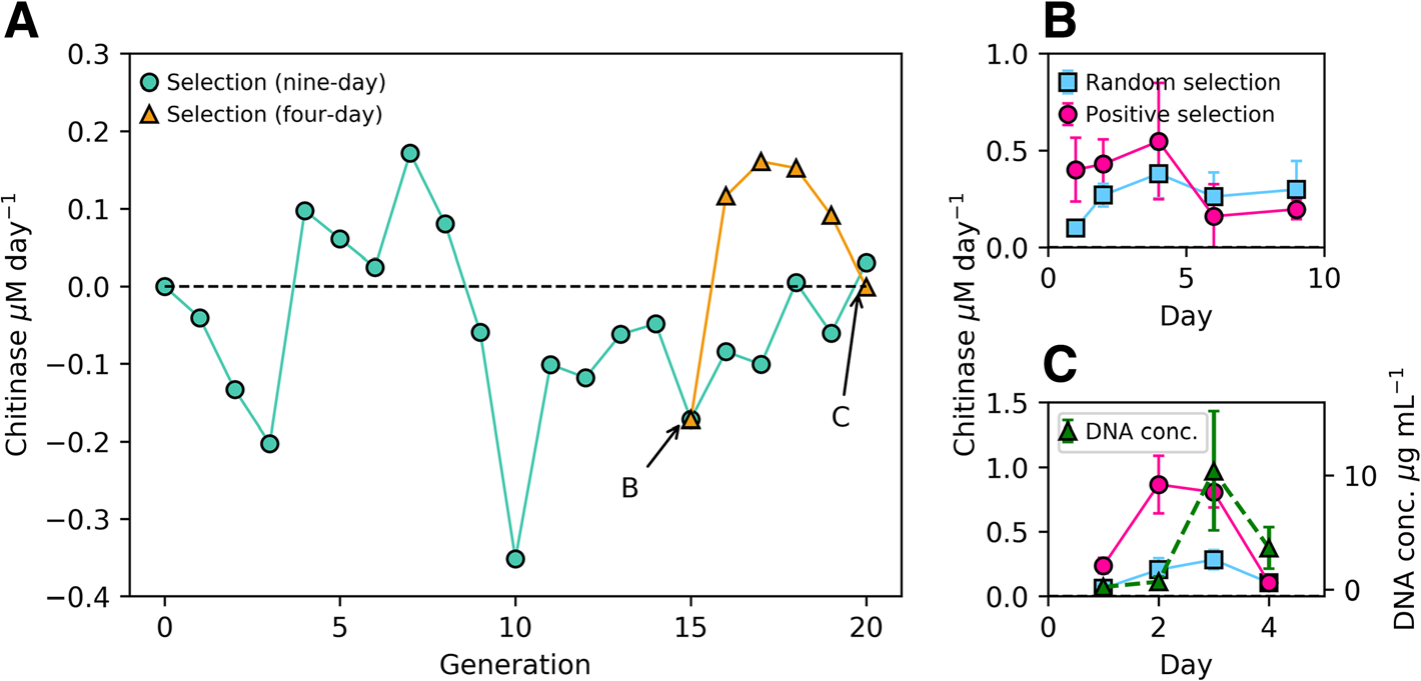
Chitinase activity in artificial selection experiment 1. **a** Enzymatic activity measured over 20 generations. Each point represents the mean of the positive selection communities (*n* = 30) from which the mean of the randomly selected controls (*n* = 30) was subtracted. The black dotted line (zero) represents where chitinase activity of the positive selection is equal to that of the random selection. **b** Chitinase activity measured within the incubation period of generation 15 of the 9-day incubation. **c** Chitinase activity and DNA concentration of the positive selection measured within the incubation period of generation 20 of the 4-day incubation. Each point in panels **b** and **c** represents absolute chitinase activity measured in the positive (red) and random selection (blue). Arrows in panel **a** indicate the generations in which the regular monitoring of chitinase activity was performed, i.e. shown in panels **b** and **c**

Our first artificial selection experiment highlighted the need to carry out each transfer when the desired trait (i.e. chitinase activity) was at its peak and not at a pre-defined incubation time, as done previously [2, 6, 8]. Initially, we set a standardised 9-day incubation time between transfers because this was the time it took for chitinase activity to peak in a preliminary enrichment experiment (data not shown). After 14 transfers, we did not observe a strong increase in chitinase activity (Fig. 2a and Additional file 1: Figure S1) and, intriguingly, in nine out of the 14 transfers, we observed a lower activity in the positive selection than in the randomly selected control (Fig. 2a), suggesting that a random selection of microcosms is more effective in enhancing chitinase activity than actively selecting for the best communities. To further investigate the reasons behind this low efficiency, we took regular enzymatic activity measurements in the incubation period between transfers 14 and 15 (Fig. 2b). We found that chitinase activity was peaking much earlier within the incubation, i.e. at day 4, and by the end of 9 days, the chitinase activity had dropped below the activities registered for the random selection experiment (Fig. 2b). Attending to this result, after transfer 15, we set up an additional experiment, run in parallel, where the incubation time between transfers was shortened to 4 days. Shortening the incubation time led to a selection of higher chitinase activities at transfers 16 and 17, but the progressive increase in activity had stalled by transfers 18 and 19 (Fig. 2a). Chitinase activity was again measured every day within the incubation period before the final transfer, 20, and we found that the enzymatic activity was almost nine times higher on day 2 than day 4 (Fig. 2c), indicating that the optimal incubation time had again been reduced. Interestingly, using DNA as a proxy for biomass (see Additional file 1: Supplementary information and Figure S2 for more details), we observed low concentrations on day 2, i.e. when chitinase activity was at its peak, indicating a very high enzymatic activity amongst the existing microbial population (Fig. 2c). Although the chitinase activity was still high on day 3, there was also a considerable increase in biomass, suggesting a relatively less-active chitinolytic community at this time point. Both chitinase activity and biomass had dropped at day 4, presumably as a consequence of grazing (as discussed below).

While the 9-day incubation experiment gave an overall negative trend, shortening the incubation times to the chitinase maxima drastically increased the benefits of artificial community selection, i.e. initially to 4 days (generation 15, Fig. 2b) and later to 2 days (generation 20, Fig. 2c). This suggests that the selection of an efficient chitin-degrading community shortens the time required not only to reach maximum chitinase activity, but also to enter decay due to community succession.

#### Microbial community succession

We carried out MiSeq amplicon sequencing of the 16S and 18S rRNA genes to characterise the microbial community succession that occurred within the first selection experiment and, by this way, gain insight into the strong variability in chitinase activity observed over time. We sequenced the communities that were used as the inoculum for each of the 20 transfers, both 9- and 4-day-long experiments, as well as the community obtained from the daily monitoring of the incubation period for transfer 20. This data was processed using both Mothur [27] and DADA2 workflows [28, 29], obtaining similar results (Additional file 1: Figures S3 and S4). DADA2 results are presented here as this workflow retains greater sequence information, better identifies sequencing errors and gives higher taxonomic resolution [30]. Unique taxa are therefore amplicon sequence variants (ASVs) rather than operational taxonomic units (OTUs).

#### Community succession over the 4-day incubation period within transfer 20

**Fig. 3:**
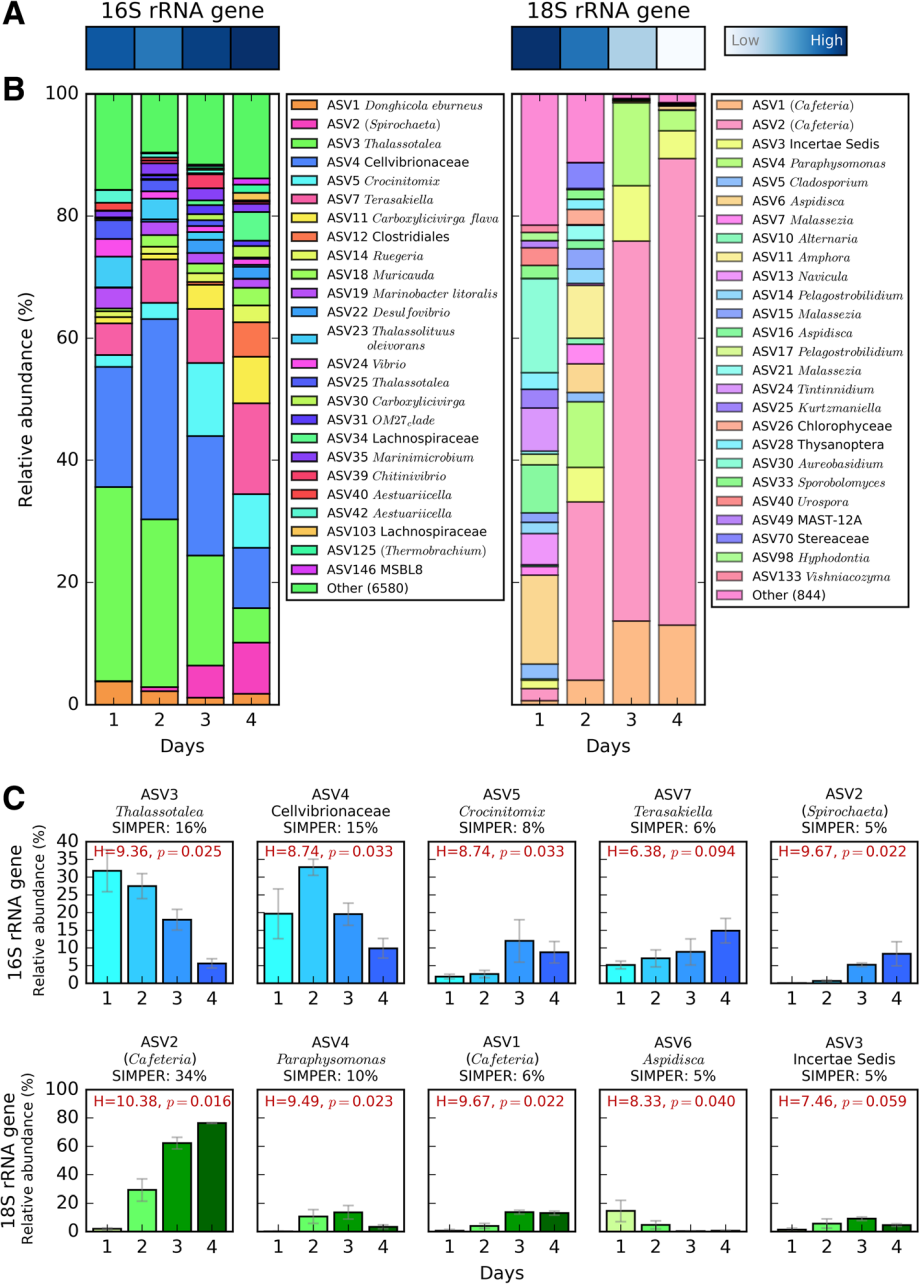
Daily microbial community analysis over the 4-day incubation period within generation 20. The analysis was performed on the three communities that showed highest chitinase activity by the end of the 4 days and which would have been used to inoculate the next generation. **a** Simpson's index of diversity of the 16S (left) and 18S rRNA gene (right) amplicon analysis. The scale ranges between 0.38 (low) and 0.93 (high). **b** Community relative abundance over the 4-day incubation period. Only ASVs with abundance above 1% in at least one time point are shown. The abundance for each ASV is a mean value from the three communities. ASVs were classified to genus level using the SILVA database (v132). Names in brackets were not identifiable with the standard analysis pipeline and were identified through a BLAST search of the NCBI database. **c** Five 16S and 18S rRNA gene ASVs that contributed the most to the community variations over time according to a SIMPER analysis. The percentage of variation to which each ASV contributes is indicated. Error bars represent the standard deviations of the three communities used to inoculate the next generation

The daily microbial community analysis over 4 days at transfer 20 showed a progressive increase in prokaryotic diversity (from 0.83 to 0.93, according to Simpsonâ€™s index of diversity) whereas a strong decrease in diversity was observed amongst the eukaryotic community (from 0.93 to 0.38; Fig. 3a). SIMPER analyses were carried out to identify those 16S and 18S rRNA gene ASVs that were contributing most to the differences over the four successive days observed in Fig. 3b. The top 5 ASVs in these analyses were responsible for 50% and 60% of the temporal variation for the 16S and 18S rRNA genes, respectively (Fig. 3c).

**Fig. 4:**
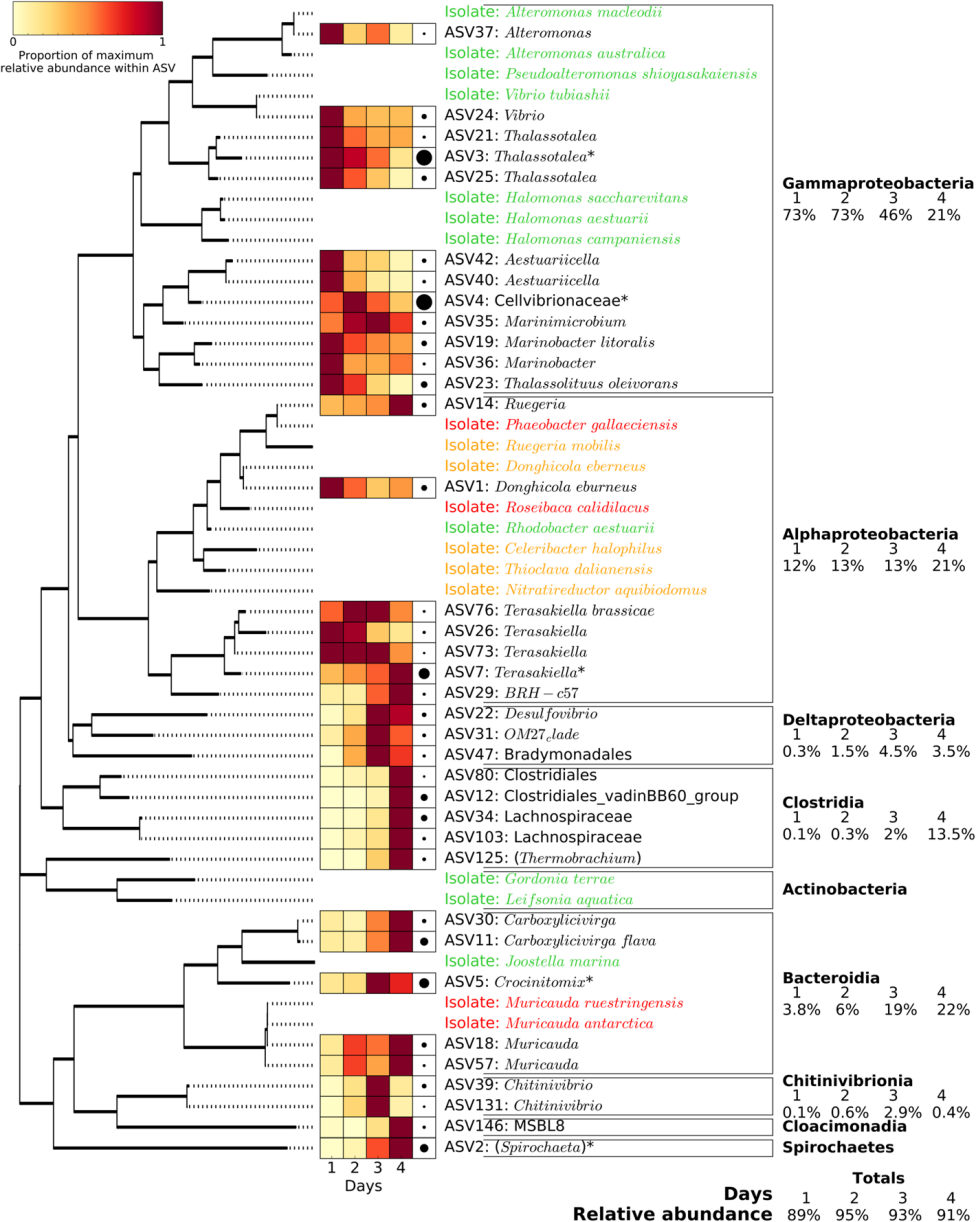
Phylogenetic analysis and relative abundance of the major 16S rRNA gene ASVs (i.e. with relative abundance above 0.5% in at least one of the 4 days) and bacterial isolates obtained at the end of the artificial selection experiment. Phylogenetic grouping is represented by a mid-point-rooted maximum likelihood phylogenetic tree. The 36 ASVs represented in the figure (out of the 6605 total ASVs detected) accounted for 92% of all 16S rRNA gene relative abundance. The heatmap represents the relative abundance of each ASV over the 4 days, with darker red showing the day at which the ASV showed maximum abundance. Black circles on the right of the heatmap represent the maximum relative abundance for that ASV amongst the entire community. The 20 isolates are coloured depending on their ability to grow on chitin and the monomer, GlcNAc (green), the GlcNAc only (orange) or neither (red)

For the 16S rRNA gene, the most important ASVs were ASV3 (*Thalassotalea*, contributing to 16% of the community variation between the four days, *p* = 0.025), ASV4 (Cellvibrionaceae, 15% variation, *p* = 0.033), ASV5 (*Crocinitomix*, 8% variation, *p* = 0.033), ASV7 (*Terasakiella*, 6% variation, *p* = 0.094) and ASV2 (*Spirochaeta*, 5% variation, *p* = 0.022) (Fig. 3c). ASVs 3 and 4 (both *Gammaproteobacteria*) represented over 50% of the prokaryotic community abundance on day 2, when chitinase activity was highest, and their abundances followed a similar pattern to the chitinase activity over 4 days (Fig. 2c), suggesting that these ASVs may be the main drivers of chitin hydrolysis. On the other hand, ASVs 7 (*Alphaproteobacteria*) and 2 (*Spirochaetes*) both showed a progressive increase over time (i.e. from a combined relative abundance of 5% on day 1 to 23% on day 4; Fig. 3c), suggesting that these ASVs could be cross-feeding organisms that benefit from the primary degradation of chitin. Interestingly, the overall 16S rRNA gene analysis also showed a strong succession over time at higher taxonomic levels (Fig. 4). While *Gammaproteobacteria* pioneered and dominated the initial colonisation and growth, presumably, via the degradation of chitin (i.e. with 73% relative abundance during the first 2 days), all other taxonomic groups became more abundant towards the end of the incubation period (e.g. *Clostridia*, *Bacteroidia* and *Alphaproteobacteria* increased from an initial relative abundance of 0.1, 2.8 and 12% on day 1 to 13.5, 22 and 21% on day 4, respectively, Fig. 4). Microbial isolates confirmed *Gammaproteobacteria* as the main contributors of chitin-biodegradation (as discussed below).

The SIMPER analysis of the 18S rRNA gene highlighted ASV2 (*Cafeteria* sp., contributing to 34% of the community variation between 4 days, *p* = 0.016), ASV4 (*Paraphysomonas*, 10% variation, *p* = 0.023), ASV1 (*Cafeteria* sp., 6% variation, *p* = 0.392), ASV6 (*Apsidica*, 5% variation, *p* = 0.040) and ASV3 (Incertae Sedis, 5% variation, *p* = 0.059) as the five main ASVs contributing to 60% of the community variation over 4 days (Fig. 3c). ASV2, which was 96% similar to the bactivorous marine flagellate *Cafeteria* sp., was by far the most striking Eukaryotic organism, showing an increase in relative abundance from 2% on day 1 up to over 76% on day 4 (Fig. 3b, c). As observed in prokaryotes, eukaryotic phylogenetic groups also showed a large variation between the beginning and the end of the incubation period, mainly due to the increase of *Bicosoecophyceae* over time (i.e. from 2.6 to 89% relative abundance driven by both ASV1 and ASV2, Additional file 1: Figure S5).

#### Community succession over the entire artificial selection experiment

**Fig. 5:**
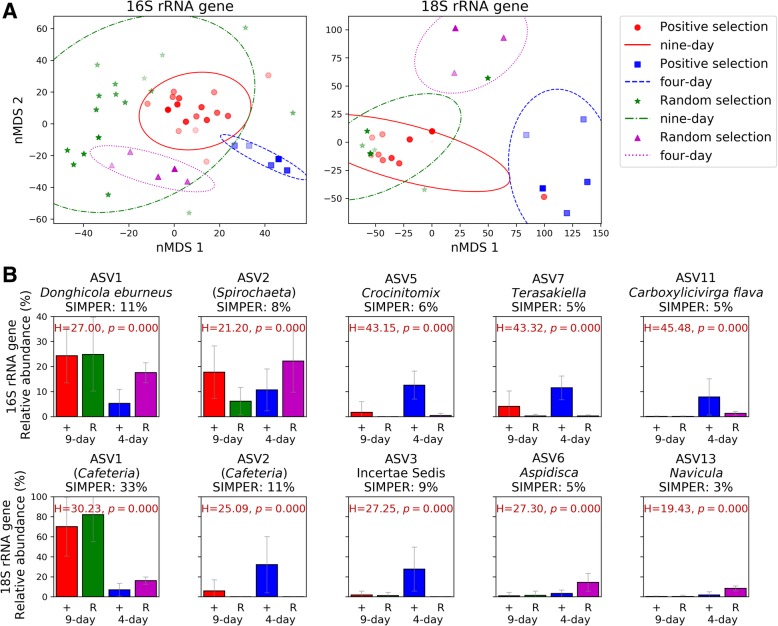
Microbial community variation over the entire artificial selection experiment. **a** nMDS plot showing Bray-Curtis distance of 16S (left) and 18S rRNA gene communities (right). Distance between the community composition obtained from 9-day (red circles) and 4-day incubations (blue squares) of the positive selection, and 9-day (green stars) and 4-day incubations (purple triangles) of the random controls are shown. The marker colour intensity correlates to the generation number, where progressive darker colours represent later generations. Each point represents the mean of the three communities selected from one generation used to inoculate the following one, for the positive selection. Random communities were pooled before sequencing. Ellipses show the mean plus the standard deviation of each group of samples. Stress values are 0.175 for the 16S rRNA gene and 0.063 for the 18S rRNA gene. **b** Five 16S (top panel) and 18S rRNA gene ASVs (bottom panel) that contributed the most towards community variations between the 9-day (generations 0â€“20) and 4-day (generations 16â€“20) positive (+) and random (R) selections according to SIMPER analyses. The percentage of variation to which each ASV contributes is indicated. ASVs were classified to the species level with the standard analysis pipeline using the SILVA database (v132) where possible. Names in brackets were not identifiable and were identified through a BLAST search of the NCBI database. Relative abundances and error bars shown are the mean and standard deviations of all generations within that treatment

We analysed the 16S and 18S rRNA gene community composition (Fig. 5, Additional file 1: Figure S6) at each transfer in order to determine the effect that positive or random selection of communities had across the 20 transfers, both for the 9-day incubation experiment (i.e. transfers 0 to 20) and shortened 4-day incubation experiment (i.e. transfers 16 to 20). Most interestingly, the overall community variability across all transfers (16S and 18S rRNA gene nMDS analysis, Fig. 5a) showed that only the positive selection of the shortened 4-day incubations differentiated the community from the random selection, which was confirmed by a PerMANOVA test using Bray-Curtis distance (16S rRNA gene *p* = 0.001, 18S rRNA gene *p* = 0.002, Additional file 1: Table S2), while the 9-day selection mostly clustered with the random control communities. This is a clear explanation as to why the 9-day incubation time was not allowing a progressive selection of a community with better chitinase activities than those obtained randomly and, only when the time was shortened, did we observe an effect of the positive selection over the random selection.

SIMPER analyses were carried out to determine the ASVs that most strongly contributed to the differences between groups (i.e. positive versus random selections and 9-day versus 4-day incubation times, Fig. 5b). For the 16S rRNA gene, the top 5 ASVs identified by the SIMPER analysis contributed to 35% of the community variation, while for the 18S rRNA gene, they accounted for 61% (Fig. 5b). The 16S rRNA gene ASVs 5, 7 and 11 (*Crocinitomix*, *Terasakiella* and *Carboxylicivirga flava*, respectively) presented a much higher abundance in the 4-day-positive selection than in any other selection (13%, 11% and 8%, respectively), suggesting that these species were the major contributors to the differentiation of these communities, as seen in Fig. 5a. As observed above for the 4-day incubation analysis, *Cafeteria* sp. (18S rRNA gene ASV1 and ASV2, both 96% similar) was again the most conspicuous eukaryotic organism. ASV2 was more abundant in the positive 4-day selection (32% of the relative abundance), while ASV1 was highest in the three other selections (70% and 82% in the positive and random 9-day selection, respectively, and 16% in the random 4-day selection; Fig. 5b).

#### Chitinase gene copies in artificially assembled metagenomes

Artificially assembled metagenomes, generated by PICRUSt [31] from the 16S rRNA gene amplicon sequences, were used to search for enzymes involved in chitin degradation: KEGG orthologs K01183 for chitinase, K01207 and K12373 for chitobiosidase, K01452 for chitin deacetylase and K00884, K01443, K18676 and K02564 for the conversion of GlcNAc to fructose-6 phosphate (Additional file 1: Figure S7 and Table S3) [32-34]. As expected from the measured chitinase activities, the shortened 4-day incubation experiment showed over 30 times more chitinase (K01183) gene copies than the 9-day incubation experiment (i.e. an average of 0.66 copies per bacterium were observed in the 4-day incubation experiment while only 0.025 copies per bacterium were observed over the same transfers in the 9-day experiment). Also, from the daily analysis of transfer 20, the chitinase activity was positively correlated with the normalised chitinase gene copy number (*r*^2^ = 0.57), with a peak in chitinase activity *and* chitinase gene copies on day 2 (i.e. over one chitinase gene copy per bacterium). The most striking result from this analysis was the strong bias of taxonomic groups that contributed to the chitinase and chitin deacetylase genes; chitinase genes were mainly detected in *Gammaproteobacteria* and some *Bacteroidia*, whereas the chitin deacetylase genes were almost exclusively present in *Alphaproteobacteria*. It is worth highlighting that the chitosanase gene (K01233), the enzyme required to hydrolyse the product from chitin deacetylation, chitosan, was not detected in any of the artificial metagenomes. Chitobiosidases (K01207 and K12373) and enzymes involved in the conversion of GlcNAc to fructose-6 phosphate (K00884, K01443, K18676 and K02564) were more widespread. Nevertheless, this data needs to be taken with caution as these were not real metagenomes.

#### Isolation and identification of chitin degraders

Bacterial isolates were obtained from the end of the artificial selection experiments to confirm the ability of the identified groups to degrade chitin. From the 50 isolates obtained, 20 were unique according to their 16S rRNA gene sequences. From these, 18 showed at least 98% similarity with one or more of the MiSeq ASVs (Additional file 1: Table S4) although, unfortunately, none belonged to the most abundant ASVs detected during the community analysis. The ability for chitin and GlcNAc degradation by each one of the isolates was assessed. We found that 16 of these isolates could grow using GlcNAc as the sole carbon source, but only 11 of these strains could grow on chitin (Fig. 4). The four remaining bacteria from the 20 isolated could not grow using chitin or GlcNAc. Most interestingly, all isolates from the class *Gammaproteobacteria* (*n* = 7) were capable of chitin degradation whereas only a smaller subset of isolates had this phenotype in other abundant taxonomic groups, such as *Bacteroidia* (1 out of 3) or *Alphaproteobacteria* (1 out of 8, Fig. 4).

We confirmed that both a cheater (i.e. isolate able to grow on GlcNAc but not chitin; *Donghicola eburneus*, *Alphaproteobacteria*) and a cross-feeder (i.e. isolate not capable of growth with GlcNAc or chitin; *Phaeobacter gallaeciensis*, *Alphaproteobacteria*) were only able to grow with chitin in the presence of a chitin-degrading isolate (*Pseudoalteromonas shioyasakaiensis*, *Gammaproteobacteria*; Additional file 1: Figure S8). As expected, while no growth was observed in the absence of the chitin degrader, both the cheater and the cross-feeder grew over two orders of magnitude more when co-cultured with the degrader (Additional file 1: Figure S8).

### Second artificial selection experiment: implementing an improved selection process

**Fig. 6:**
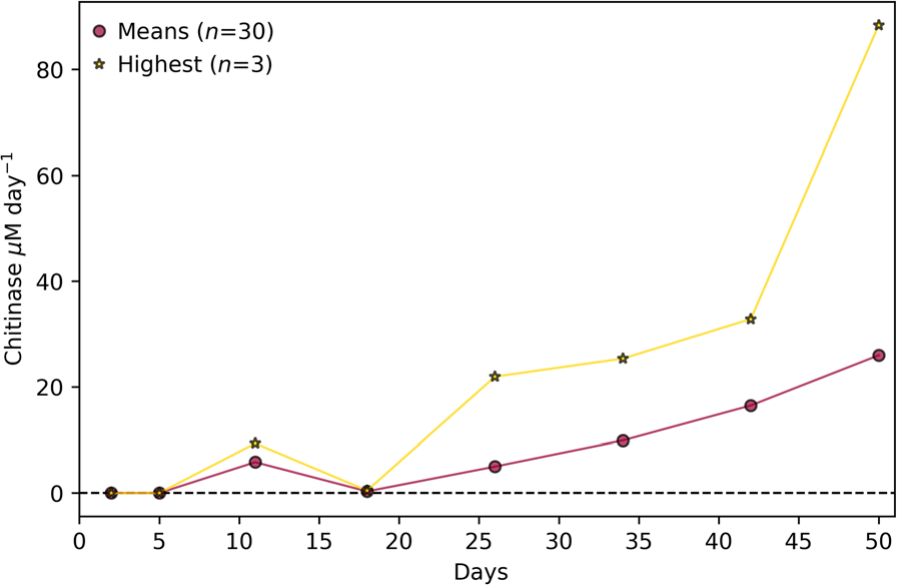
Chitinase activity of artificial selection experiment 2. The graph shows the mean chitinase activity of the positive selection, from which the mean of the random selection was subtracted. The means of all communities within the generation (*n* = 30; red) and those of only the three communities that were pooled for the inoculum of the next generation (yellow) are shown

A second selection experiment showed an extremely rapid boost in chitinase activity, demonstrating that implementing an optimised incubation time between transfers largely enhances the selection of a desired trait. For this experiment, chitinase activity was measured daily until a peak in chitinase activity was observed. The communities with the highest chitinase activity on this day were used to transfer to the next set of microcosms. By implementing this improved technique, we measured chitinase activity of almost 90 µM day^-1^ in only 7 transfers (Fig. 6 and Additional file 1: Figure S9), when the maximum activity achieved in the first experiment was 0.9 µM day^-1^ (Fig. 2c). While the different culture conditions between both experiments may have exacerbated the differences (i.e. the first artificial selection was carried out in 22 mL vials, with intermittent shaking and incubated at 23 °C, and the second artificial selection was carried out in 2% mL 96-well plates, with constant shaking and incubated at 30 °C), the fact that the randomly selected control from the second artificial selection experiment reached similar chitinase activity levels to those observed in the first experiment (i.e. ~ 0.08 μM day^-1^, Additional file 1: Figure S9) suggests the culture conditions were not the underlying reason behind the strong increase in chitinase activity observed during the second experiment where the conditions were optimised.

## Discussion

Artificial selection of microbial communities is, in principle, a powerful and attractive technique which has surprisingly been used in only a limited number of studies to date [2, 6, 8], possibly due to the lack of success as a consequence of poor process optimisation. Here, using chitin degradation as a case study and a detailed analysis of the community succession, we show that artificial selection of microbial communities can be greatly improved by controlling the incubation times between transfers. We believe that the rapid succession of microbial community structure means transfers need to be done at the peak of the selected phenotypic activity (e.g. chitinase activity) or these get swiftly replaced by less efficient communities of cross-feeding microorganisms (i.e. â€˜cheatersâ€™ and grazers). Previous studies that have artificially selected microbial communities for a particular phenotype did not report optimisation of the incubation time between transfers [2, 6, 8] which, in our hands, would have resulted in a negative selection (Fig. 2). In agreement with our results, Penn and Harvey [9] suggested that the observed phenotype in artificial ecosystem selection experiments could be significantly affected by interactions between different species and therefore microbial community structure.

A comprehensive understanding of microbial ecology helps to explain the importance of the timing during transfers. Datta et al. [10] observed three distinct stages of community structure during the colonisation of chitin particles: (a) attachment, (b) selection and (c) succession. Each phase was characterised by having relatively higher abundances of organisms that were (a) good at attaching to chitin particles, (b) good at degrading chitin particles and (c) not able to degrade chitin, but able to benefit from others that could, i.e. cheaters and cross-feeders [17, 18, 22, 35]. During our first experiment, as communities became better and faster at degrading chitin, we were measuring the chitinase activity when the communities were in the succession rather than in the selection stage and, by that point, the active chitinolytic community had decayed and was dominated by a cross-feeding community (Figs. 3 and 4). Hence, it was only when selecting at phenotypic time optima when chitinase activity improved and the overall community differentiated from the random control communities (Figs. 5 and 6). Due to the stochasticity of complex microbial communities, this time optima is difficult to predict and continuous phenotypic monitoring is required. It is also interesting to note the selection of the grazer *Cafeteria* sp. (90% of the eukaryotic community), a genus of bactivorous marine flagellates that are commonly associated with marine detritus [36]. The predator-prey dynamics postulated by Lotka, Volterra's equations would also support the need to shorten transfer times to favour the prey's growth, i.e. chitinolytic bacteria [37, 38]. We note, however, that there may be a lower limit to optimal transfer times (possibly around 2, 3 days, Fig. 2c) as sufficient time has to be given for slower growing marine taxa [39] to (i) develop a biofilm [40], (ii) initiate the hydrolysis of the polymer to access the sugars and (iii) allow the generation of sufficient biomass to overcome the dilution between transfers.

Interestingly, a strong successional pattern was observed at a higher taxonomic level. While *Gammaproteobacteria* dominated during the initial stages when chitinase activity was at its peak (accounting for over 70% of the prokaryotic community), other groups increased in abundance during the later stages (i.e. *Alphaproteobacteria*, *Bacteroidia* and *Clostridia*), similarly to the pattern previously observed by Datta et al. [10] and Enke et al. [22, 35]. The fact that *Gammaproteobacteria* are major contributors to chitin degradation is not new [41-46]. All *Gammaproteobacteria* isolates obtained from the end of the experiments were able to grow using chitin as the only source of carbon and energy (Fig. 4) confirming that this class is likely responsible for most of the chitinase activity observed. On the other hand, *Alphaproteobacteria*, the numerically dominant class of heterotrophic bacteria in surface oceans [47, 48], follow a cross-feeding and/or cheating life-strategy as five out of eight *Alphaproteobacterial* isolates could only use *N*-acetyl-d-glucosamine (GlcNAc) and only one could use chitin (Additional file 1: Table S4). The dependence of cheaters and cross-feeders on the presence of a chitin-degrader was confirmed with co-cultures (Additional file 1: Figure S8) and agreed with the results generated by others [22].

The PICRUSt metagenome analysis (Additional file 1: Figure S7) further confirmed that almost all chitinase gene copies were encoded by *Gammaproteobacteria* (i.e*.* 90%; almost one gene copy encoded per bacterium) and, to a lesser extent, by some *Bacteroidia*. Chitin is made up of molecules of GlcNAc linked by (1,4)-Î²-glycosidic bonds, and it has previously been found that initial degradation of chitin takes place predominantly by (i) chitinases which depolymerise the (1,4)-Î²-glycosidic bonds either at the ends or in the middle of chains, or (ii) chitobiosidase enzymes which also hydrolyse (1,4)-Î²-glycosidic bonds but only at the ends of chitin chains. Genes for the intracellular enzymes involved in GlcNAc utilisation (i.e. the transformation of GlcNAc to fructose-6-phosphate) were much more widespread amongst different taxonomic groups, highlighting the broader distribution of cross-feeding or cheating organisms which can benefit from the extracellular depolymerisation of chitin which generates freely available GlcNAc to the community. Alternative degradation of chitin may also occur by deacetylation and deamination of the GlcNAc amino sugar, transforming chitin into chitosan and cellulose, respectively, after which they can be depolymerised by a range of other enzymes (e.g. chitosanases or cellulases) [26, 49, 50]. While *Alphaproteobacteria* did not contribute to chitinase enzymes, they *did* potentially encode for most of the chitin deacetylases in the system, although no chitosanases were detected.

Chitinolytic organisms have previously been found to make up between 0.1 and almost 6% of prokaryotic organisms in aquatic ecosystems [17, 51], while over a third of the organisms in these habitats can utilise only the products of chitin hydrolysis (i.e. GlcNAc) [17, 52-54]. With *Gammaproteobacteria* being primarily responsible for the degradation of chitin here, the success of the artificial selection for an enhanced chitinolytic community was possibly achieved by the selective enrichment of this group between the beginning (5% of the prokaryotic community, within the expected range of *Gammaproteobacteria,* found within natural environments) [17, 51] and end of the experiment (75% of the community).

Finally, chitin degradation is a task that single microorganisms can perform efficiently, but other more laborious phenotypic traits are rarely carried out entirely by a single microorganism in nature. It is now well documented that a distribution of labour is favoured in natural microbial communities [55-59]. The detrimental effects of community dynamics and drift described here could be overcome by synthetically assembled microbial communities, preventing the system from moving away from the high-performing desired community. Nevertheless, this still requires a comprehensive understanding of the community structure and the necessity to select and isolate the microbes of interest.

## Conclusions

Here, we have proven the validity of artificially selecting a natural microbial community to better degrade chitin, but have highlighted the caveats for achieving this goal, which require a better understanding of the ecology of the system. We found that continuous optimisation of incubation times is essential in order to successfully implement this process, as optimal communities rapidly decay due to their replacement by cheaters and cross-feeders, as well as the increase of potential predators such as grazers and, although not tested here, viruses. Hence, future artificial selection experiments should adjust transfer incubation times to activity maxima to successfully evolve enhanced community phenotypes and, eventually, allow the enrichment and isolation of microbes of interest.

## Materials and methods

### Microbial inoculum

The microbial community used as an inoculum was obtained from bulk marine debris collected during boat tows from both Plymouth Sound (Devon, UK; June 2016) and Portaferry (Northern Ireland, UK; August 2016).

### Chitinase activity measurements

Chitinase activity was measured as the liberation of the fluorescent molecule 4-methylumbelliferyl (MUF) from three chitinase substrates (MUF-*N*-acetyl-β-d-glucosaminide, MUF-β-d-*N*,*N*’-diacetylchitobioside and MUF-β-d-*N*,*N*’,*N*’’-triacetylchitotrioside; Sigma Aldrich, UK), following the previously described method [42, 60, 61] (Additional file 1: Supplementary information). Standards curves were obtained using chitinase from *Streptomyces griseus* (Sigma Aldrich, UK) dissolved in sterile phosphate-buffered saline solution (pH 7.4; 0.137 M) with a highest concentration of 0.1 UmL^-1^ (activity equivalent to 144 μM day^-1^). Samples were diluted prior to measurement if they were expected to be above this range.

### Artificial selection

The process for artificial selection is depicted in Fig. 1. Briefly, 30 individual microcosms per treatment and generation were incubated in the dark under the conditions described below. At the end of each incubation period, the three microcosms with the highest chitinase activities (or three random microcosms in the case of the control) were pooled and used as the inoculum for the next generation of microcosms (*n* = 30). This was repeated across multiple transfers. Two artificial selection experiments were performed, the first to optimise the process and the second to implement optimal conditions and achieve a high-performing chitinolytic microbial community.

#### First artificial selection experiment

Incubations were carried out at 23 °C in 22 mL glass vials (Sigma Aldrich), each containing 20 mL of autoclaved seawater (collected from outside Plymouth Sound, Devon, UK; June 2016) supplemented with NaH_2_PO_4_, F/2 trace metals [62] (Additional file 1: Supplementary information) and 100 mg of chitin powder (from shrimp shells; Sigma Aldrich) as the sole source of carbon and nitrogen. Generation 0 was started with 200 μL of microbial inoculum. The efficiency of the selection process was assessed by comparing a ‘positive selection’ (where the three communities with highest activity were pooled and 200 μL was used to inoculate each one of the 30 microcosms of the next generation) against a ‘random selection’ (where three communities were chosen at random, using a random number generator within the Python module Random, to inoculate the following generation) to give a control against uncontrollable environmental variation [63]. Each treatment was repeated across 20 generations with incubation times of 9 days. In parallel, treatments where incubation times were shortened to 4 days were set up after generation 15. Samples were taken from each community and stored in 20% glycerol at , 80 °C for further microbial isolation, and pellets from 1.5 ml of culture were collected by centrifugation (14,000 x *g* for 5 min) and stored at ,-20 °C for final DNA extraction and community analysis.

#### Second artificial selection experiment

A second selection experiment was set up implementing optimal transfer incubation times. Microcosms were incubated in 2 mL 96-well plates (ABgene^TM^, ThermoFisher Scientific) covered by Corning® Breathable Sealing Tapes to stop evaporation and contamination while allowing gas exchange. Each well contained 1.9 mL of a custom mineral media containing MgSO_4_, CaCl_2_, KH_2_PO_4,_ K_2_HPO_4_, 0.52 M NaCl and artificial seawater trace metals (Additional file 1: Supplementary information), supplemented with 10 mg of chitin powder. The microbial inoculum was 100 μL (i.e. initial inoculum and transfer between generations). Chitinase activity was measured daily. Transfer between generations was carried out just after the peak of chitinase activity had occurred, calculated as the mean chitinase activity across the 30 microcosms of the positive selection treatment. Plates were incubated in the dark at 30°C with constant shaking (150rpm). Eight days was the maximum incubation time allowed to reach maximum chitinase activity due to volume constraints. Pellets from 1.5 mL were collected by centrifugation (14,000 x *g* for 5 min) and stored at -20°C for final DNA extraction

### DNA extraction and amplicon sequencing

DNA was extracted using the DNeasy Plant Mini Kit (Qiagen) protocol, with modifications as follows (adapted from [64]): 300 μL 1x TAE buffer was used to resuspend cell pellets and these were added to ~ 0.4 g of sterile 0.1 mm BioSpec Zirconia/Silica Beads in 2 mL screw cap microtubes (VWR international). Bead beating was carried out for 2 x 45 s and 1 x 30 s at 30 Hz using a Qiagen Tissue Lyser. Cell lysates were then processed in accordance with the manufacturer's instructions, with an extra centrifugation step to ensure all liquid was removed (1 min, 13,000 x *g*) directly before elution of samples. A Qubit® HS DNA kit (Life Technologies Corporation) was used for DNA quantification after which they were diluted to equalise the concentrations across samples. A Q5® Hot Start High-Fidelity 2X Master Mix (New England Biolabs® Inc.) was used to amplify the 16S rRNA gene v4-5 regions using primers 515F-Y and 926R [65], and the 18S rRNA gene v8, 9 regions using primers V8F and 1510R [66] (Additional file 1: Supplementary information). PCR products were purified using AmpliClean Magnetic Beads (NimaGen, The Netherlands). Index PCR was carried out using Illumina Nextera Index Kit v2 adapters. Samples were normalised using a SequelPrep^TM^ Normalisation Plate Kit (ThermoFisher Scientific). Samples were pooled and 2 x 300 bp paired-end sequencing was carried out using the MiSeq system with v3 reagent kit. Negative DNA extraction controls and library preparation negative controls as well as chitin-only positive controls were processed and sequenced alongside samples.

### Microbial community structure determination

Two different workflows were used to analyse the sequencing data: DADA2 [28, 29] and Mothur [27]. DADA2 delivers better taxonomic resolution than other methods (e.g. Mothur) as it retains unique sequences and calculates sequencing error rates rather than clustering to 97% similarity [30]. The resultant taxonomic units are referred to as amplicon sequence variants (ASVs) rather than operational taxonomic units (OTUs from Mothur). For the DADA2 analysis, sequencing data were processed following the DADA2 (version 1.8.0) pipeline [28]. Briefly, the data were filtered, i.e. adapter, barcode and primer clipped, and the ends of sequences with high numbers of errors were trimmed. The amplicons were denoised based on a model of the sequencing errors and paired-end sequences were merged. Only sequences between 368, 379 for the 16S rRNA gene and 300, 340 for the 18S rRNA gene were kept and chimaeras were removed. The resulting ASVs were classified using the SILVA reference database (v132) [67]. For the Mothur analysis [27], sequencing data were filtered, i.e. adapter, barcode and primer clipped, sequence length permitted was 450 bp for the 16S rRNA gene and 400 bp for the 18S rRNA gene, maximum number of ambiguous bases per sequence = 4 and maximum number of homopolymers per sequence = 8. Taxonomy assignment was performed using the SILVA reference database (Wang classification, v128) [67] and operational taxonomic units (OTUs) set at 97% similarity. For both processing workflows, chloroplasts, mitochondria and Mammalia were removed from the 16S rRNA gene and 18S rRNA gene datasets; eukaryotes were removed from the 16S rRNA gene dataset; and bacteria and archaea were removed from the 18S rRNA gene dataset. The average number of reads per sample was approximately 12,500 for the 16S rRNA gene and 20,000 (Mothur) or 34,000 (DADA2) for the 18S rRNA gene. Samples with less than 1000 total reads were excluded from downstream analyses. Although most analyses were carried out using relative abundance, each sample was subsampled at random to normalise the number of reads per sample, and the resulting average coverage was 92% (Mothur) or 94% (DADA2) for the 16S rRNA gene and 99% (Mothur and DADA2) for the 18S rRNA gene.

### Microbial isolation and characterisation

Microbes were isolated from the final transfer of positive selection experiments by plating serial dilutions on Marine Broth 2216 (BD Difco^TM^) and mineral medium plates (i.e. custom medium, Additional file 1: Supplementary information) supplemented with 0.1% *N*-acetyl-d-glucosamine (GlcNAc) and 1.5% agar. Colonies were re-streaked on fresh agar plates until pure isolates were obtained. The identification of isolates was carried out by sequencing the partial 16S rRNA gene (GATC BioTech, Germany) using primers 27F and 1492R [68] (Additional file 1: Supplementary information).

Isolates were grown in custom mineral medium supplemented with either 0.1% chitin or 0.1% GlcNAc (*w*/*v*), as sources of carbon and nitrogen, to test for chitinase activity and chitin assimilation, respectively. Growth was monitored over 14 days by measuring (i) chitinase activity (as described above), (ii) optical density at 600 nm and (iii) protein content (following the manufacturer's instructions; QuantiPro^TM^ BCA Assay Kit, Sigma Aldrich, UK). Isolates were also tested on custom mineral medium agar plates made with the addition of 0.1% chitin and 0.8% agarose. Plates were incubated at 30°C for 21 days to allow the formation of halos indicative of chitinase activity.

Co-cultures were performed using isolates *Pseudoalteromonas shioyasakaiensis* (chitin degrader), *Donghicola eburneus* (cheater, capable of growth on GlcNAc but not chitin) and *Phaeobacter gallaeciensis* (cross-feeder, not capable of growth on either chitin or GlcNAc). Combinations of these strains were grown in 25 cm^2^ tissue culture flasks with 25 mL custom mineral media (Additional file 1: Table S1) supplemented with 0.1% (*w*/*v*) of chitin. Cultures were incubated at 30 °C with shaking at 200 rpm for 3 days. Pellets from 1.5 ml of culture on days 0 and 3 were collected by centrifugation (14,000 x *g* for 5 min) and stored at , -20°C for DNA extraction, as above. Specific primers were designed for each of the isolates (see Additional file 1: Supplementary methods and materials), and qPCR was performed (Applied Biosystems 7500 Fast Real-Time PCR system) using 1 μL template DNA following the manufacturer's instructions for the GoTaq® qPCR Master Mix (Promega). Final primer concentrations were 0.5, 0.9 and 0.9 M, for the degrader, cheater and cross-feeder, respectively. Results were normalised to standard curves that used DNA extracted from pure cultures.

### Statistical analyses

All analyses of chitinase activity and most MiSeq data analyses were carried out using custom Python scripts (Python versions 2.7.10 and 3.6.6) using the modules colorsys, csv, heapq, matplotlib, numpy, os, pandas, random, scipy, scikit-bio, sklearn [69] and statsmodels. SIMPER analyses and plotting of phylogenetic trees were performed in R (R version 3.3.3) [70] using the following packages: ape [71], dplyr, ggplot2, gplots, ggtree [72], lme4, phangorn [73], plotly, tidyr, vegan [74] and phyloseq [75]. The top 5 ASVs identified in each SIMPER analyses were classified to their closest relative using a BLAST search of the GenBank database. Hypothetical community functions were obtained using PICRUSt in QIIME1 [31, 76] by mapping ASVs to the Greengenes database [77] (v13.5) at the default 97% similarity threshold. The PICRUSt analysis includes almost 35% of all ASVs, accounting for a mean relative abundance of 53%, 68% and 81% for the positive selection 9-day, 4-day and daily analyses, respectively. The Nearest Sequenced Taxon Index (NSTI) obtained for each of the taxonomic groups is available in Additional file 1: Table S3 (a full summary for each sample can be found on GitHub https://github.com/R-Wright-1/ChitinActivity). The three groups with the highest relative abundance, *Gammaproteobacteria*, *Alphaproteobacteria* and *Bacteroidia* (i.e. 29.3%, 26.4% and 16.6%, respectively) showed NSTI values of 0.07, 0.09 and 0.18, respectively. Sequences used for phylogenetic trees were aligned using the SILVA Incremental Alignment (www.arb-silva.de) [78] and mid-point rooted maximum likelihood trees were constructed using QIIME1 [76]. All scripts can be found at  https://github.com/R-Wright-1/ChitinActivity. All sequences have been deposited in the NCBI Short Read Archive (SRA) database under Bioproject PRJNA499076. qPCR data was analysed using custom Python scripts.

## Additional file

Additional file 1: Supplementary information and supplementary materials and method: **Tables S1**, **S4** and **Figures S1**, **S9**. (DOCX 1240 kb)

## Data Availability

All sequences have been deposited in the NCBI Short Read Archive (SRA) database under Bioproject PRJNA499076. All additional data and script files used for analysis can be found at https://github.com/R-Wright-1/ChitinActivity. Data will be uploaded to a data repository if this manuscript is accepted.
