# Supplementary material for: Understanding microbial community dynamics to improve optimal microbiome selection
Source: Microbiome. 2019 Jun 3;7:85. doi: 10.1186/s40168-019-0702-x (PMC6547603; doi:10.1186/s40168-019-0702-x)
Supplement: Supplementary file 1 — Supplementary information and supplementary materials and method: Tables S1, S4 and Figures S1, S9. (DOCX 1240 kb) [file 40168_2019_702_MOESM1_ESM.docx]

**Supplementary information**

**Understanding microbial community dynamics to improve optimal microbiome selection**

Robyn Wright^1^, Matthew Gibson^2,3^ and Joseph A. Christie-Oleza^1*^

^1^ School of Life Sciences, University of Warwick, Coventry, UK

^2^ Department of Chemistry, University of Warwick.

^3^ Medical School, University of Warwick, Coventry, UK

*corresponding authors at: School of Life Sciences, University of Warwick, Coventry CV4 7AL, UK.

[J.Christie-Oleza@warwick.ac.uk](mailto:J.Christie-Oleza@warwick.ac.uk). Tel: 024 765 72969

**Supplementary materials and methods**

*Chitinase activity measurement*

Chitinase activity was measured as the liberation of the fluorogenic molecule 4-methylumbelliferyl (MUF) from three chitinase substrates, following the methods of Hood (1991), Lecleir *et al.* (2007) and Köllner *et al.* (2012). These were the chitinase substrates specific to the monomer, dimer and trimer; MUF-N-acetyl-β-D-glucosaminide, MUF-β-D-N,N’-diacetylchitobioside and MUF-β-D-N,N’,N’’-triacetylchitotrioside, respectively. Stock solutions of each substrate were prepared in 100% dimethyl formamide. These three MUF substrates were combined in equal concentrations so as the test substrate had a concentration of 100 µM of each. MUF hydrolysis assays were performed in a black 96-well polypropylene microplate. 36 µL sample was added to 4 µL 100 µM MUF, and these were incubated at room temperature (~20˚C) for 1 hour. The reaction was stopped with 160 µL 100 mM glycine-sodium hydroxide buffer (pH 10.4). Fluorescence was measured with 362 nm excitation and 448 nm emission using a Synergy HTX microplate reader. Fluorescence measurements were converted U ml^-1^ enzyme activity, where 1 U is the amount of enzyme required to catalyse the conversion of 1 µM of substrate to product per minute. This used standard solutions made with chitinase from *Streptomyces griseus* (~9 U mg^-1^) dissolved in sterile phosphate buffered saline solution (pH 7.4) with a highest concentration of 0.1 U ml^-1^. The volume of sample taken for chitinase activity measurements was the same for all experiments. Chitinase activity was calculated as a function of µmoles of chitin consumed per litre and day (µM day^-1^). The growth of biofilms is notoriously difficult to measure [4], and we were therefore unable to normalise chitinase activity measurements to biomass. We did attempt to normalise to protein content measurements (Fig. S2), however, the standard curve was not consistent for measurements taken in seawater, and no consensus could be reached for different dilutions of the same samples. The presence of chitin particles interferes with any absorbance-based method (as also noted by Datta *et al.* (2016) in [5]) and accurate methods for the quantification of biofilms generally requires microscopy [6,7], thereby making it difficult to carry out in a high throughput manner, as would have been necessary for this study. Extracted DNA concentration is therefore shown along with absolute chitinase activity measurements in Figs. S1 and S9. See Mandakhalikar *et al.* (2017) for a summary of current methods for biofilm quantification and [8] Azeredo *et al.* (2017) [7] for a detailed and critical overview.

*Media preparation*

For the first artificial selection experiment, additional nutrients and trace metals, as in F/2 medium (Table S1) [9], were added to seawater (collected from Plymouth Sound, Devon, UK; June 2016). For the second experiment, custom mineral media nutrients and trace metals were added to MilliQ water along with 3% (w/v) NaCl (Table S1). Vitamins were not added to the media used for the artificial selection experiment. All media was autoclaved before use.

**Table S1.** Details of concentrations of F/2 and custom mineral media nutrients and trace metals.

| **Media** | **Nutrients** | **Final concentration (M)** |
| --- | --- | --- |
| F/2 | $\mathrm{Na}H_{2}\mathrm{PO}_{4}\cdot2H_{2}O$ | 3.62 x 10^-5^ |
| Custom mineral media | $\mathrm{MgS}O_{4}\cdot5H_{2}O$  $\mathrm{Ca}\mathrm{Cl}_{2}\cdot2H_{2}O$  $KH_{2}PO_{4}$  $K_{2}\mathrm{HP}O_{4}$ | 2 x 10^-3^  2 x 10^-4^  7 x 10^-3^  6 x 10^-3^ |
|  | **Trace metals** | **Final concentration (M)** |
| F/2 | $\mathrm{Fe}\mathrm{Cl}_{3}\cdot6H_{2}O$ | 1.17 x 10^-5^ |
|  | $\mathrm{Na}_{2}EDTA\cdot2H_{2}O$ | 1.17 x 10^-5^ |
|  | $\mathrm{Cu}\mathrm{SO}_{4}\cdot5H_{2}O$ | 3.93 x 10^-8^ |
|  | $\mathrm{Na}_{2}\mathrm{Mo}O_{4}\cdot2H_{2}O$ | 2.6 x 10^-8^ |
|  | $\mathrm{ZnS}O_{4}\cdot7H_{2}O$ | 7.65 x 10^-8^ |
|  | $\mathrm{Co}\mathrm{Cl}_{2}\cdot6H_{2}O$ | 4.2 x 10^-8^ |
|  | $\mathrm{Mn}\mathrm{Cl}_{2}\cdot4H_{2}O$ | 9.1 x 10^-7^ |
| Custom mineral media | $H_{3}\mathrm{BO}_{3}$  $\mathrm{Mn}\mathrm{Cl}_{2}\cdot4H_{2}O$  $\mathrm{ZnS}O_{4}\cdot7H_{2}O$  $\mathrm{Na}_{2}\mathrm{Mo}O_{4}\cdot2H_{2}O$  $\mathrm{Cu}\mathrm{SO}_{4}\cdot5H_{2}O$  $\mathrm{Co}\left( NO_{3} \right)_{2}\cdot6H_{2}O$  $\mathrm{Fe}\mathrm{Cl}_{3}\cdot6H_{2}O$  $\mathrm{EDTA}\left( \mathrm{Na}_{2}\mathrm{Mg} \right)$ | 4.6 x 10^-4^  1.4 x 10^-4^  1.4 x 10^-3^  1.9 x 10^-6^  5 x 10^-9^  2.7 x 10^-8^  1.8 x 10^-6^  7.1 x 10^-6^ |
|  | **Vitamins^1^** | **Final concentration (M)** |
|  | $C_{12}H_{18}\mathrm{Cl}_{2}N_{4}\mathrm{OS}$ (Thiamine HCl) | 2.9 x 10^-5^ |
|  | $C_{6}H_{5}NO_{2}$ (Nicotinic acid) | 1.6 x 10^-4^ |
|  | $C_{8}H_{12}\mathrm{ClN}O_{3}$ (Pyridoxine HCl) | 9.7 x 10^-5^ |
|  | $C_{7}H_{7}NO_{2}$ (Para-aminobenzoic acid) | 73 x 10^-5^ |
|  | $C_{17}H_{20}{N_{4}O}_{6}$ (Riboflavin) | 5.3 x 10^-5^ |
|  | $C_{10}H_{16}N_{2}O_{3}S$ (Biotin) | 4 x 10^-6^ |
|  | $C_{63}H_{88}\mathrm{Co}N_{14}O_{14}P$ (Cyanocobalamin vitamin B12) | 7.4 x 10^-7^ |

^1^ In the isolate co-culture experiments 0.005% yeast extract was used in place of the vitamins.

*Primers used for sequencing and qPCR*

For MiSeq amplicon sequencing, 16S rRNA gene (V4) primers 515F-Y (GTGYCAGCMGCCGCGGTAA) and 926R (CCGYCAATTYMTTTRAGTTT) [10] and 18S rRNA gene (V8-9) primers V8F (ATAACAGGTCTGTGATGCCCT) and 1510R (CCTTCYGCAGGTTCACCTAC) [11] were used to give amplicon sizes of approximately 372 and 375 bp, respectively.

For Sanger sequencing, 16S rRNA gene primers 27F (AGAGTTTGATCMTGGCTCAG) and 1492R (TACGGYTACCTTGTTACGACTT) [12] were used to give a product of approximately 1450 bp. For qPCR to determine the abundance of the three selected isolates grown in co-cultures, primers were designed based on the 16S rRNA gene sequences to avoid cross-hybridization. For *Pseudoalteromonas shioyasakaiensis*, the forward (CAACAGTTGGAAACGACTGC) and reverse (ATCGTCGCCTTGGTGAGCCA) primers give a product size of 134 bp, for *Donghicola eberneus* the forward (AATAGTCCCGGGAAACTGGG) and reverse (ATCGTAGACTTGGTAGGCCA) primers give a product size of 122 bp and for *Phaeobacter gallaeciensis* the forward (AATAGCCACTGGAAACGGTG) and reverse (ATCGTAGACTTGGTAGGCCG) primers give a product size of 122 bp.

**Supplementary results**

| 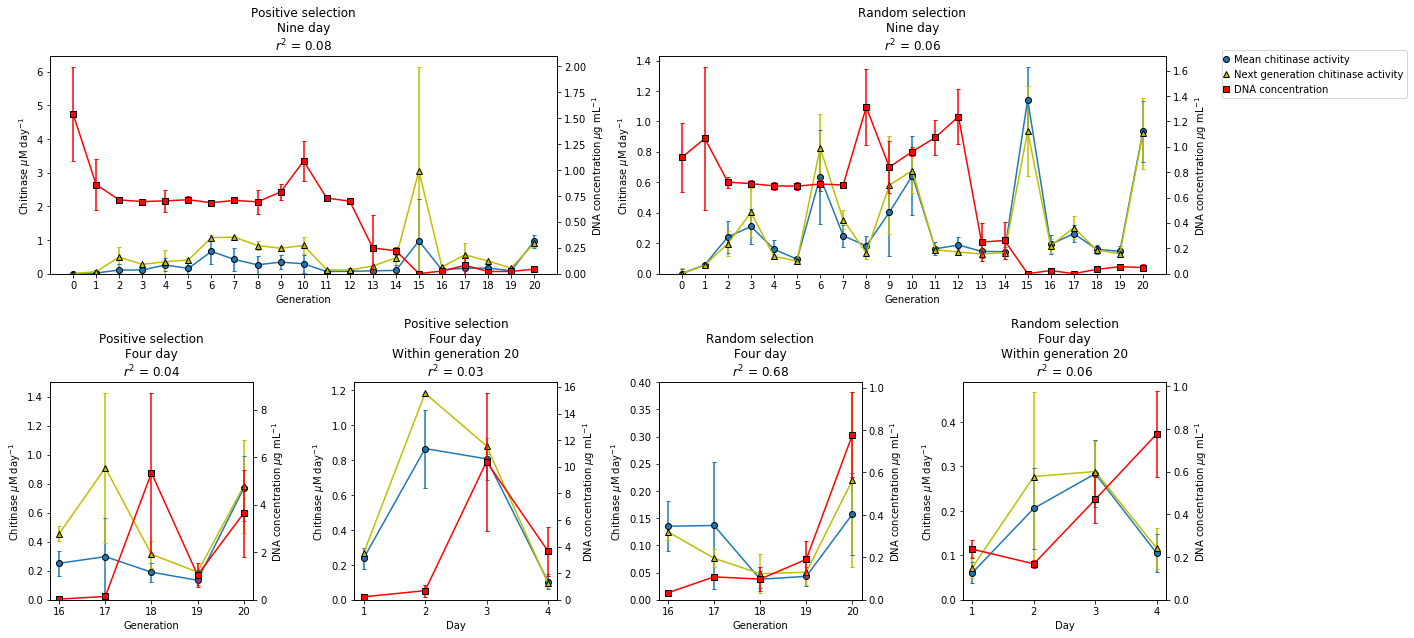 |
| --- |
| **Figure S1**. Averaged absolute chitinase activity measured from all communities (*n*=30; blue lines with circular markers) within each generation, and from the three selected communities used to inoculate the following generation (yellow lines with triangular markers) during artificial selection experiment 1. Red lines with square markers show DNA concentrations for the three communities used to inoculate the next generation and used for 16S rRNA gene amplicon sequencing. Error bars show standard deviations. Note that scales differ between all axis. *r^2^* values indicate Pearson’s correlation coefficients between chitinase activity (next generation) and DNA concentration. |

| *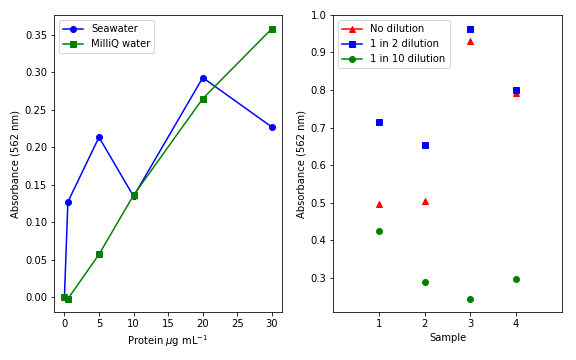* |
| --- |
| **Figure S2**. Protein content measurements using the BCA QuantiPro Assay Kit for a standard curve using either autoclaved seawater or MilliQ water (left) and four samples at three dilutions each (right). |

| 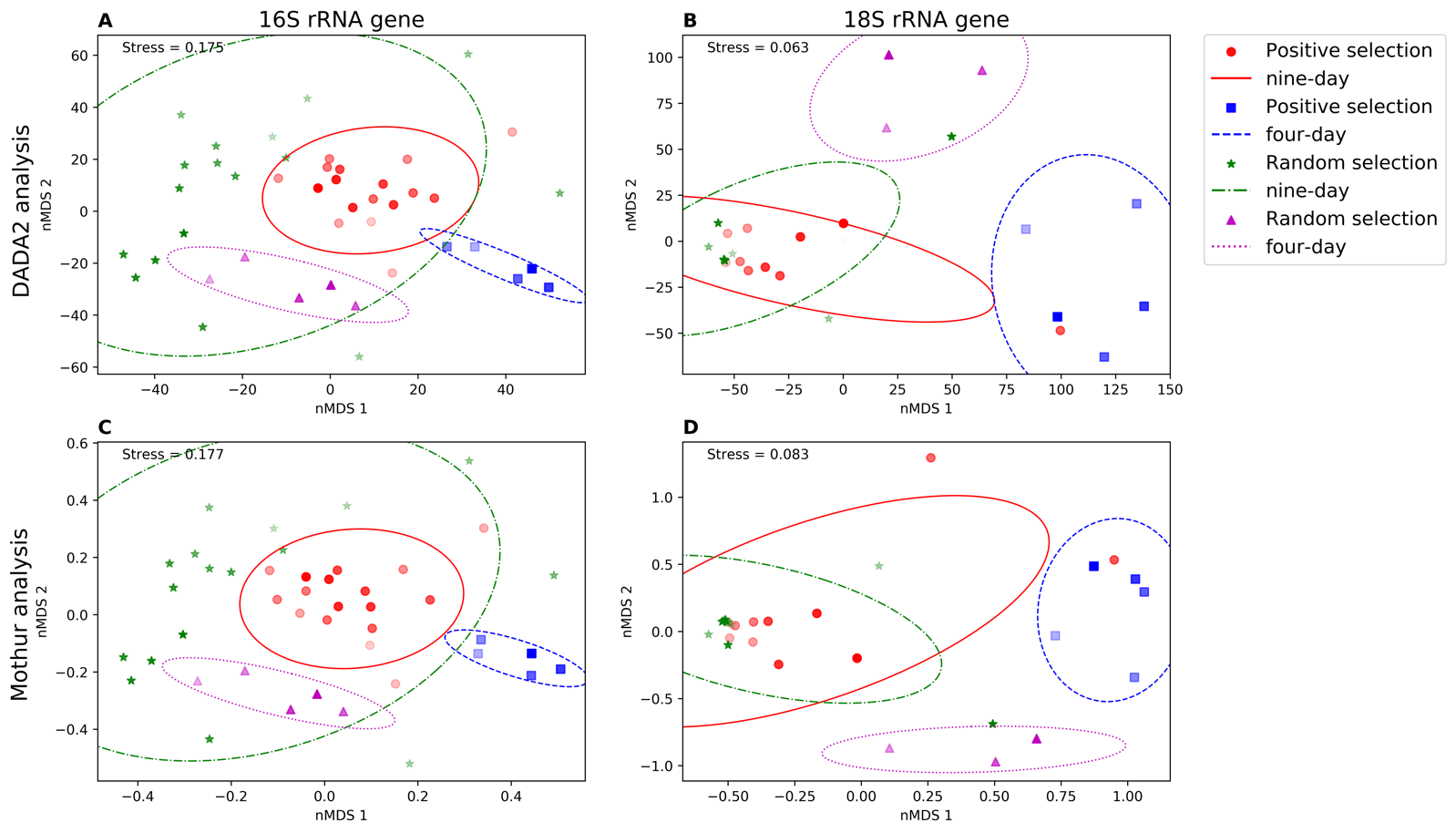 |
| --- |
| **Figure S3**. Comparison of the microbial community variation over the entre artificial evolution experiment as shown by the DADA2 (top) and Mothur (right) analysis pipelines. nMDS plots showing Bray-Curtis distance of the 16S rRNA gene and 18S rRNA gene communities for the DADA2 (panels A and B) and Mothur (panels B and C) analyses. Distance between the community composition obtained from nine-day (red circles) and four-day incubations (blue squares) of the positive selection, and nine-day (green stars) and four-day incubations (purple triangles) of the random controls are shown. Marker colour intensity correlates to generation number, where progressive darker colours represent later generations. Each point represents the mean of the three communities selected from one generation used to inoculate the following one. Ellipses show the mean plus the standard deviation of each group of samples. |

| 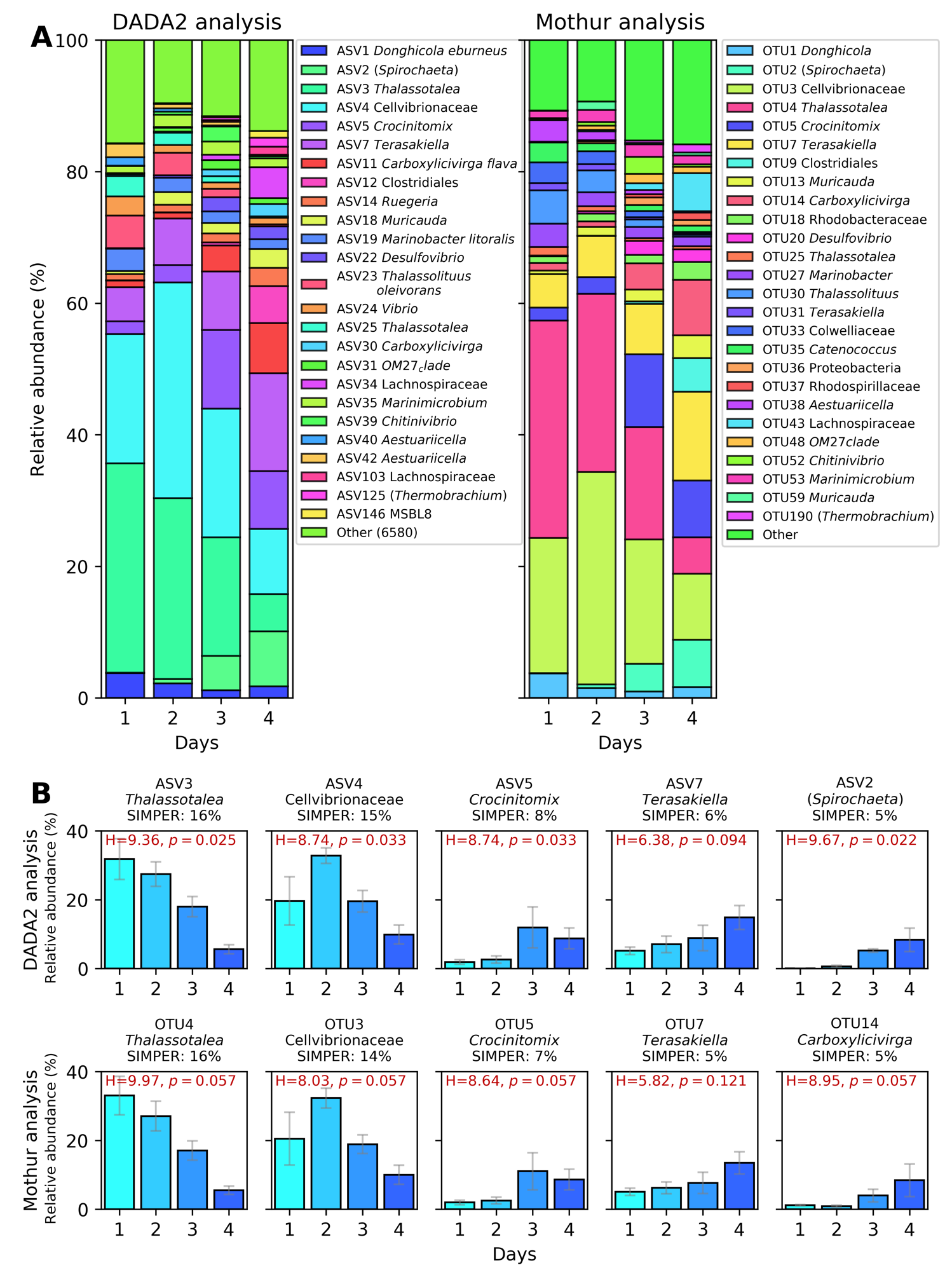  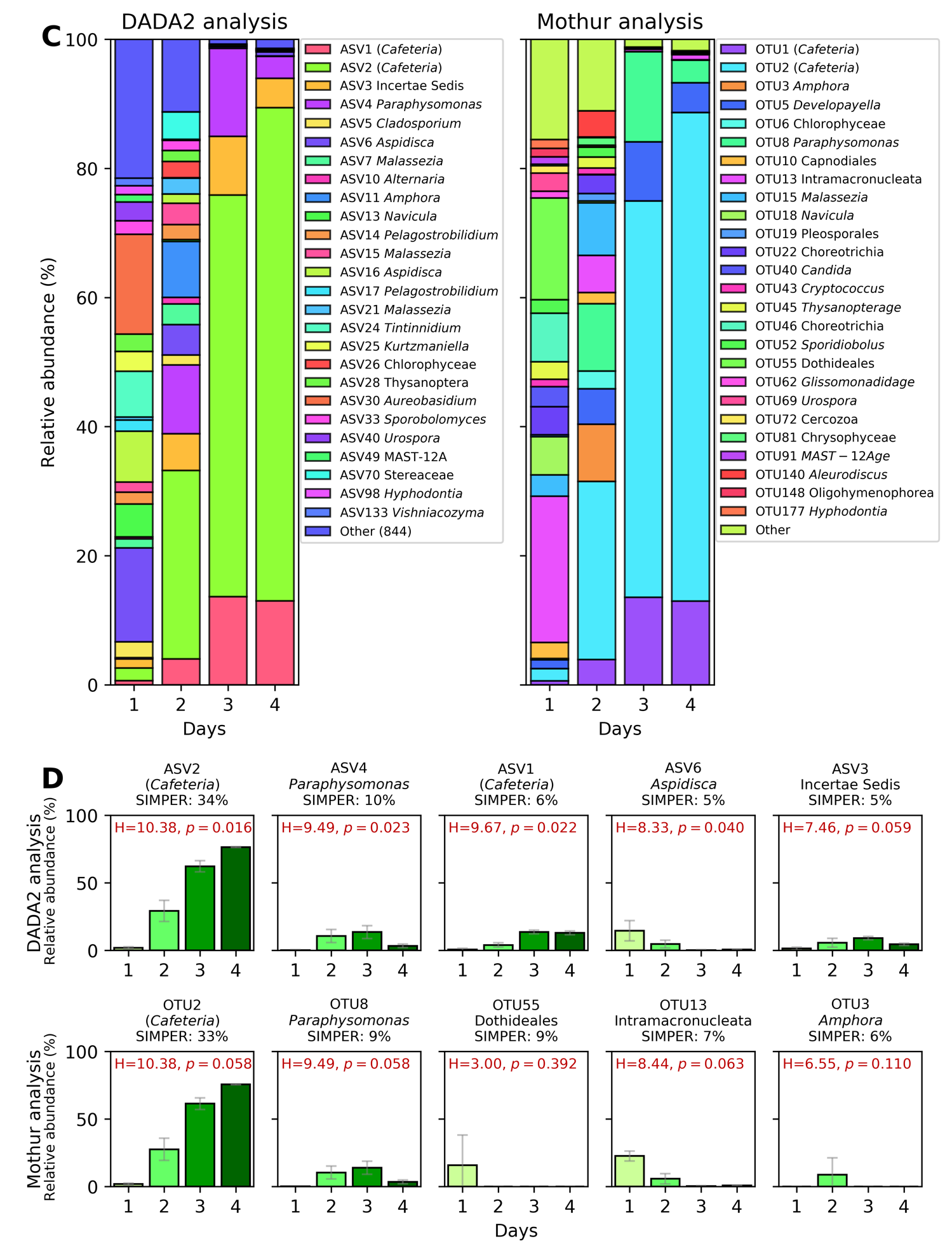 |
| --- |
| **Figure S4**. Comparison between the DADA2 and Mothur analyses for the 16S (**A** and **B**) and 18S (**C** and **D**) rRNA genes over the four-day incubation period within generation 20. (**A** and **C**) Community relative abundance over the four-day incubation period. Only ASVs with abundance above 1% in at least one time point are shown. The abundance for each ASV/OTU is a mean value of three communities used to inoculate the next generation.  ASVs/OTUs were classified to genus level by SILVA. Names in brackets were not identifiable with the standard analysis pipeline and were identified through a BLAST search of the NCBI database. (**B** and **D**) Five ASVs/OTUs that contributed the most to the community variations over time according to a SIMPER analysis. The percentage of variation to which each ASV/OTU contributes is indicated. Error bars represent the standard deviations of three communities used to inoculate the next generation. |

|  |
| --- |
| **Figure S5**. Phylogenetic analysis and relative abundance of the major 18S rRNA gene ASVs (*i.e.* with relative abundance above 0.5% in at least one of the four days). Phylogenetic grouping is represented by a mid-point rooted maximum likelihood phylogenetic tree. The 43 ASVs represented in the figure (out of the 870 total ASVs detected) accounted for 95% of all 18S rRNA gene relative abundance. The heatmap represents the relative abundance of each ASV over the four days, being the darker red the day at which the ASV showed maximum abundance. Black circles on the right of the heatmap represents the maximum relative abundance for that ASV amongst the entire community. |

| 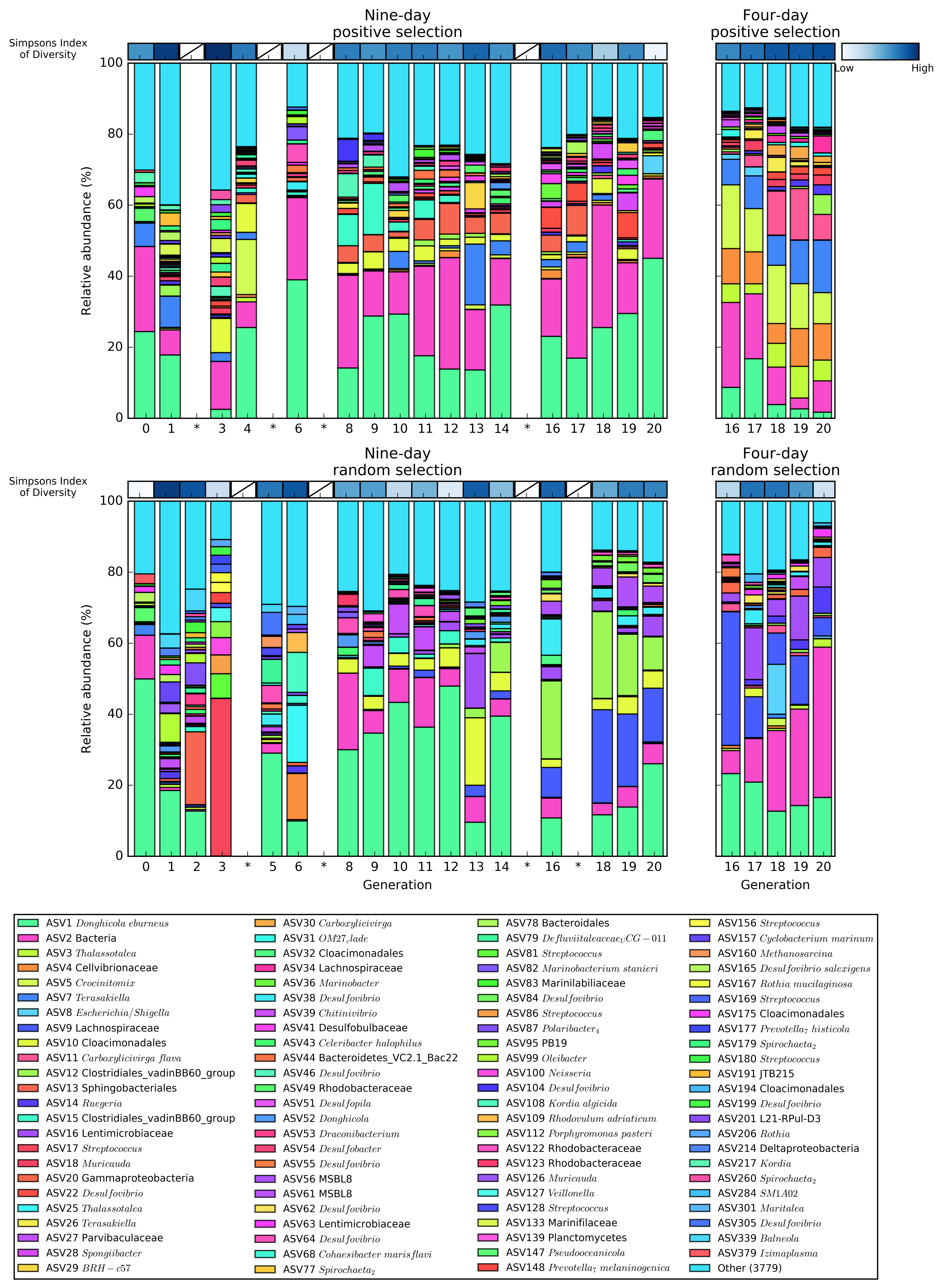  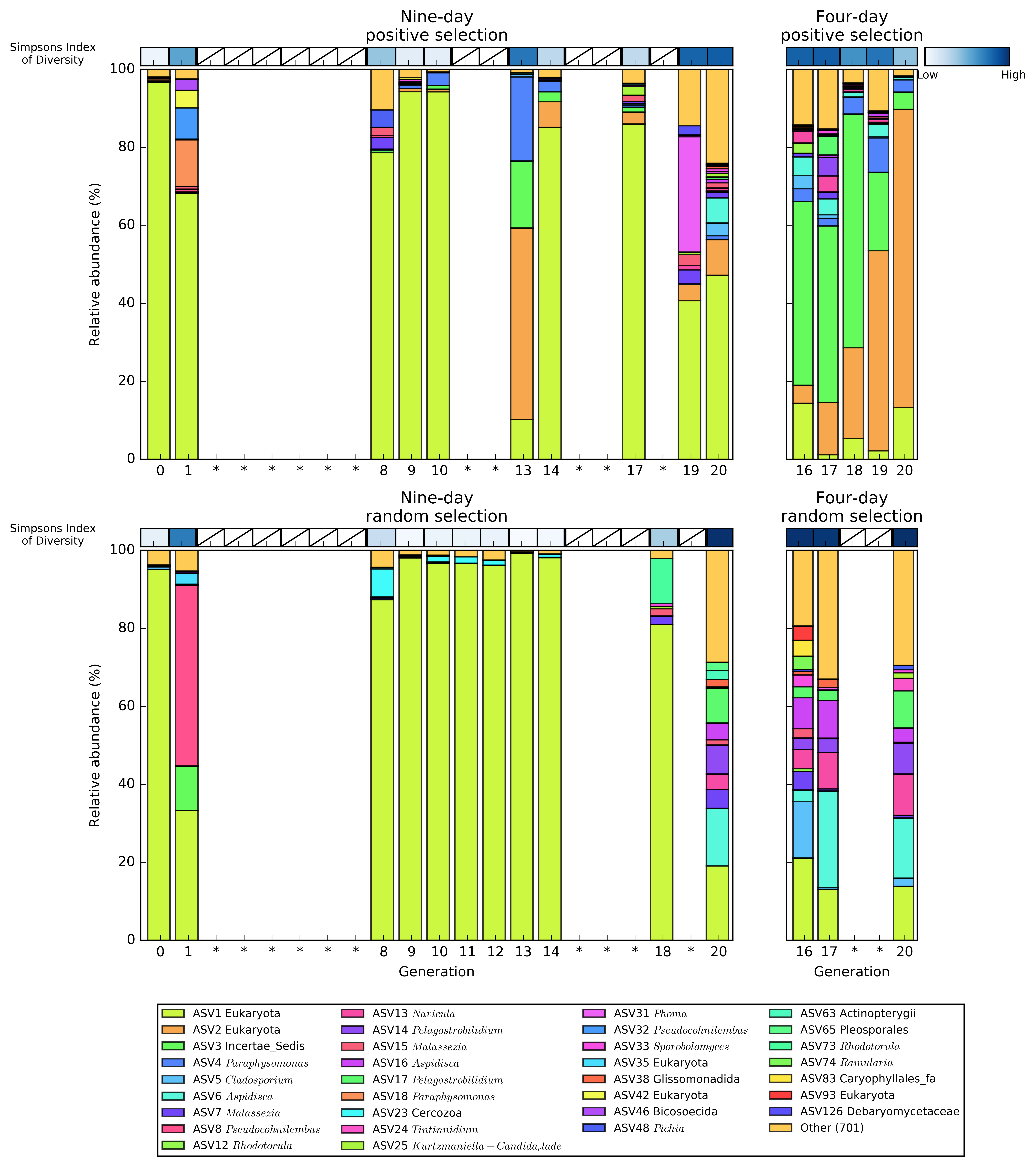 |
| --- |
| **Figure S6**. 16S **(A)** and 18S **(B)** rRNA gene community analysis of each generation within the artificial evolution experiment, both positive (top panel) and random selection (bottom panel). Simpsons index of diversity is represented above each panel, ranging between 0.73 (low diversity, white) to 0.96 (high diversity, dark blue) for the 16S rRNA gene and 0.015 (low diversity, white) to 0.92 (high diversity, dark blue) for the 18S rRNA gene. The stacked bar charts represent the relative abundance of those ASVs with abundances above 2% in at least one sample. All low abundance ASVs (3,779 and 701 ASVs for the 16S and 18S rRNA genes, respectively) are grouped into the category ‘Other’. Each bar chart is the mean value of the three communities that were pooled together for inoculating the following generation. Asterisks indicate those generations where the number of reads were too low (*i.e.* below 1,000) and these samples were removed. |

**Table S2**. Values for PerMDISP, PerMANOVA and ANOSIM statistical tests between the positive and random selections for both nine- and four-day incubation times. Controls include chitin only and negative DNA extraction controls as well as negative PCR controls.

| **Comparison** | | **PerMDISP** | | **PerMANOVA** | | **ANOSIM** | |
| --- | --- | --- | --- | --- | --- | --- | --- |
|  |  | F | p | Pseudo-F | p | R | p |
| **Positive vs random (four-day)** | 16S | 0.0105 | **0.909** | 16.7 | **0.001** | 0.960 | **0.001** |
|  | 18S | 1.006 | **0.321** | 4.91 | **0.002** | 0.638 | **0.006** |
| **Positive vs random (nine-day)** | 16S | 4.004 | 0.043 | (6.69)* | (0.001)* | (0.430)* | (0.001)* |
|  | 18S | 1.000 | **0.355** | 1.204 | 0.232 | -0.023 | 0.532 |
| **Samples vs controls (all)** | 16S | ﻿0.161 | **0.797** | ﻿0.905 | **0.002** | ﻿4.883 | **0.001** |
|  | 18S | ﻿0.0001 | **0.991** | ﻿0.732 | **0.001** | ﻿5.338 | **0.002** |

*Tests were not statistically valid due to significant PerMDISP result.

| **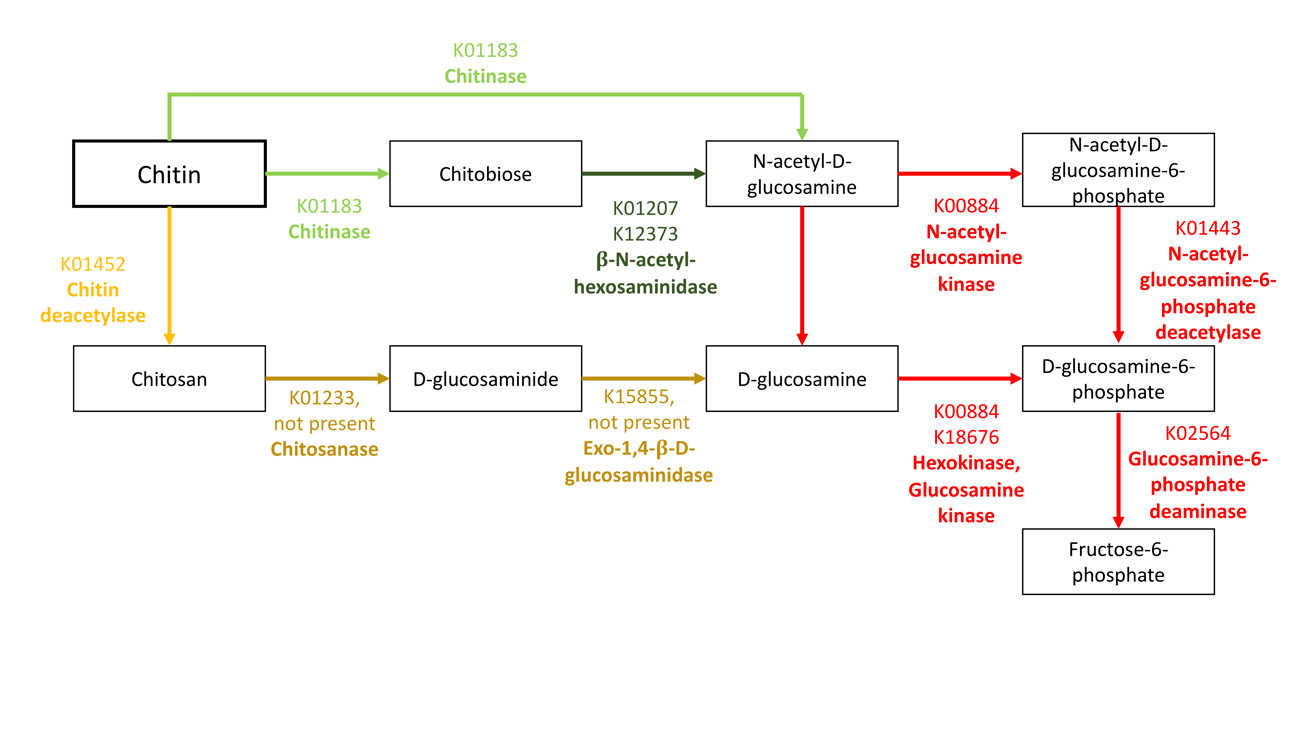**  **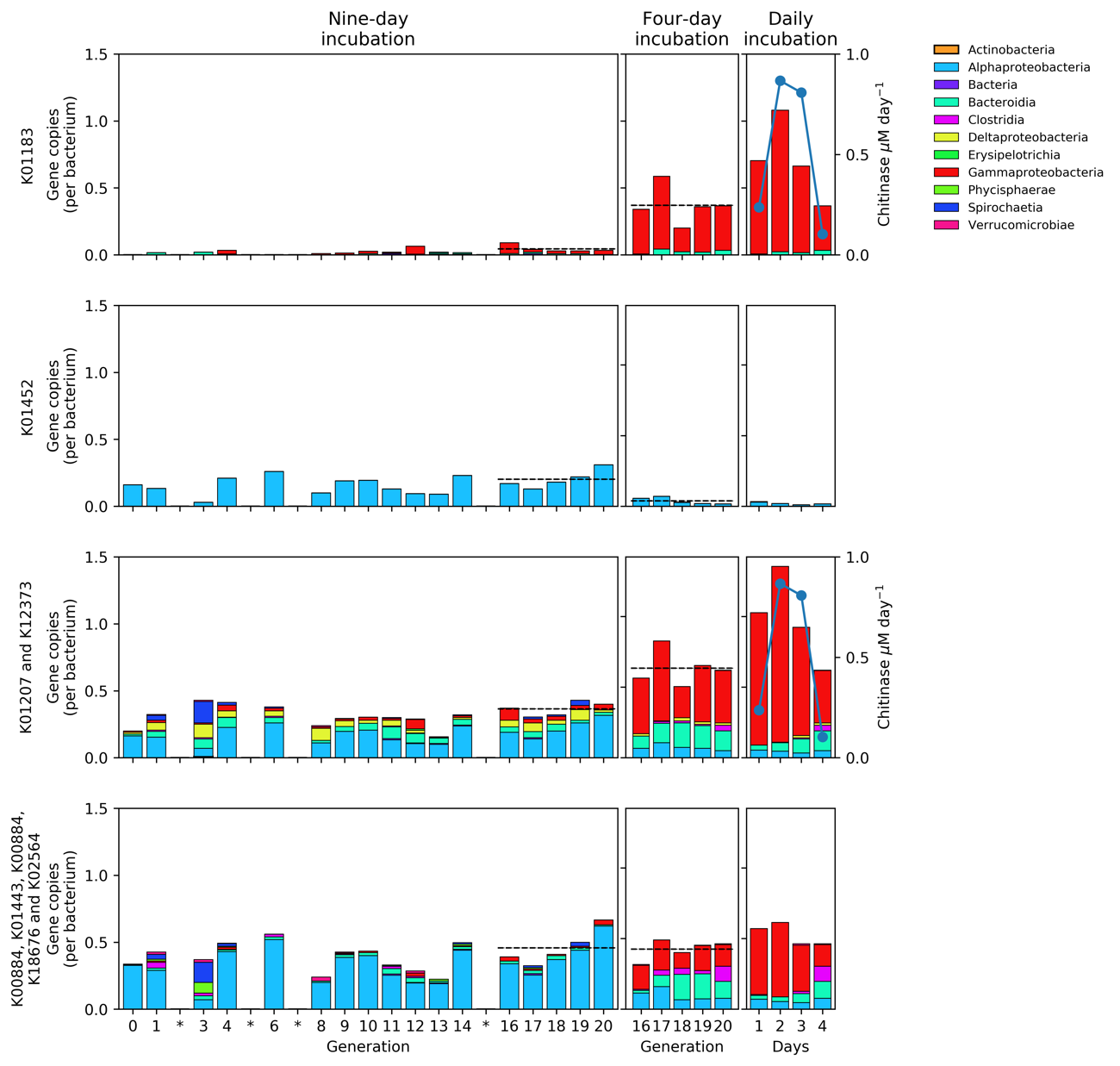** |
| --- |
| **Figure S7**. Chitin degradation pathway and the enzymes involved (adapted from KEGG pathway KO00520 for amino sugar and nucleotide sugar metabolism), showing KEGG orthologs that were searched for in the PICRUSt artificial metagenome (top). The PICRUSt analysis includes almost 35% of all ASVs, accounting for a mean relative abundance of 53%, 68% and 81% for the nine-day, four-day and daily analyses, respectively. Only the positive selection of generations 0-20 of the nine-day incubation analysis and generations 16-20 from the shortened four-day incubations are shown. The daily analysis within generation 20 of the four-day incubation is also shown. The blue points are for chitinase activity during the daily analysis of generation 20, and are shown in the panels where the KEGG orthologs would be able to produce a chitinase activity signal using the enzyme assay. Dashed black lines show means of gene copy numbers in generations 16-20. Copies of KEGG orthologs were predicted separately for each taxonomic class. Generations with asterisks indicated sample that were removed due to the low number of reads. |

**Table S3.** Summary of the information obtained from the PICRUSt analyses. The full table can be found with the data and script files on Github.

| **Class^1^** | **ASVs predicted^2^** | **Total ASVs^3^** | **Relative abundance original (%)^2^** | **Relative abundance in PICRUSt analyses (%)^3^** | **Mean weighted NSTI (not including nan)** |
| --- | --- | --- | --- | --- | --- |
|  |  |  |  |  |  |
| Acidimicrobiia | 11 | 14 | 0.00 | 0.00 | 0.23 |
| Actinobacteria | 120 | 130 | 0.06 | 0.06 | 0.05 |
| Alphaproteobacteria | 361 | 1387 | 26.39 | 15.24 | 0.09 |
| Anaerolineae | 15 | 28 | 0.08 | 0.00 | 0.31 |
| Babeliae | 0 | 16 | 0.00 | 0.00 |  |
| Bacilli | 94 | 100 | 0.10 | 0.09 | 0.04 |
| Bacteroidia | 504 | 994 | 16.55 | 14.65 | 0.18 |
| Campylobacteria | 15 | 23 | 0.23 | 0.23 | 0.03 |
| Chitinivibrionia | 0 | 12 | 0.92 | 0.00 |  |
| Chlamydiae | 2 | 12 | 0.00 | 0.00 | nan |
| Cloacimonadia | 35 | 170 | 2.40 | 1.47 | 0.17 |
| Clostridia | 232 | 517 | 4.56 | 3.18 | 0.18 |
| Deltaproteobacteria | 163 | 607 | 6.08 | 3.14 | 0.06 |
| Erysipelotrichia | 10 | 13 | 0.02 | 0.02 | 0.06 |
| Gammaproteobacteria | 478 | 1280 | 29.28 | 28.18 | 0.07 |
| Gracilibacteria | 3 | 37 | 0.01 | 0.00 | 0.56 |
| Ignavibacteria | 3 | 41 | 0.24 | 0.00 | 0.36 |
| Microgenomatia | 10 | 71 | 0.01 | 0.00 | 0.77 |
| Mollicutes | 5 | 124 | 0.23 | 0.00 | 0.12 |
| NA | 19 | 440 | 11.64 | 0.22 | 0.36 |
| Negativicutes | 21 | 23 | 0.01 | 0.01 | 0.06 |
| Other | 0 | 205 | 0.21 | 0.00 |  |
| Parcubacteria | 1 | 119 | 0.02 | 0.00 | 0.81 |
| Phycisphaerae | 10 | 23 | 0.10 | 0.10 | 0.57 |
| Planctomycetacia | 12 | 22 | 0.00 | 0.00 | 0.22 |
| Saccharimonadia | 13 | 30 | 0.02 | 0.01 | 0.46 |
| Spirochaetia | 26 | 68 | 0.59 | 0.33 | 0.18 |
| Verrucomicrobiae | 34 | 51 | 0.12 | 0.12 | 0.16 |
| Woesearchaeia | 0 | 48 | 0.11 | 0.00 |  |
| **TOTALS** | **2197** | **6605** | 100.00 | 67.04 |  |

^1^ PICRUSt was run separately for each taxonomic class.

^2^ The number of ASVs that were able to be matched to the Greengenes reference database (v13.5) prior to running PICRUSt.

^3^ The number of ASVs from that taxonomic class in the full MiSeq amplicon sequencing results.

**Table S4.** Information on isolates obtained from generation 20.

| **Species match (%)^1^** | **ASV most similar to^2^** | **Chitin/GlcNAc growth^3^** | **Other information** |
| --- | --- | --- | --- |
| *Joostella marina* (100%) | ASV1137 (90%) | C+ G+ | N-acetylglucosamine not utilised as sole carbon source, chitin not tested [13] |
| *Muricauda antarctica* (97%) | ASV57 (99%) | -- | Positive for N-acetyl-$\beta$-glucosaminidase, chitin not tested [14] |
| *Muricauda ruestringensis* (96%) | ASV18 (100%) | -- | No report of GlcNAc or chitin testing [15] |
| *Gordonia terrae* (99%) | ASV3386 (99%) | C+ G+ | No report of GlcNAc or chitin testing. Genus is metabolically diverse [16] |
| *Leifsonia aquatica* (99%) | ASV3205 (99%) | C+ G+ | No report of GlcNAc or chitin testing [17] |
| *Phaeobacter gallaeciensis* (100%) | ASV14 (100%) | -- | No report of GlcNAc or chitin testing [18] |
| *Ruegeria mobilis* (99%) | ASV88 (100%) | G+ | Not able to degrade chitin, GlcNAc not tested [19] |
| *Donghicola eberneus* (99%) | ASV1 (99%) | G+ | Negative for N-acetyl-$\beta$-glucosaminidase, chitin not tested [20] |
| *Roseibaca calidilacus* (98%) | - | -- | No available information |
| *Rhodobacter aestuarii* (98%) | ASV3991 (99%)  ASV1 (98%) | C+ G+ | No report of GlcNAc or chitin testing [21] |
| *Celeribacter halophilus* (99%) | ASV43 (99%) | G+ | Previously Huaishuia halophila [22]. Negative for N-acetyl-$\beta$-glucosaminidase, chitin not tested [23] |
| *Thioclava dalianensis* (100%) | ASV1566 (100%)  ASV0043 (95%) | G+ | Positive for N-acetylglucosamine utilisation, chitin not tested [24] |
| *Nitratireductor aquibiodomus* (98%) | ASV1008 (97%)  ASV14 (90%) | G+ | Positive for N-acetyl-$\beta$-glucosaminidase [25] |
| *Alteromonas macleodii* (99%) | ASV37 (99%) | C+ G+ | No report of GlcNAc or chitin testing [26] |
| *Alteromonas australica* (97%) | ASV321 (99%)  ASV37 (98%) | C+ G+ | Weakly utilises N-acetylglucosamine, chitin not tested [27] |
| *Pseudoalteromonas shioyasakaiensis* (99%) | ASV72 (99%) | C+ G+ | Produces N-acetyl-$\beta$-glucosaminidase, chitin not tested [28] |
| *Vibrio tubiashii* (100%) | ASV6516 (100%)  ASV24 (100%) | C+ G+ | Chitin hydrolysed extracellularly, GlcNAc not tested [29] |
| *Halomonas saccharevitans* (98%) | ASV59 (100%) | C+ G+ | No report of GlcNAc or chitin testing [30] |
| *Halomonas aestuarii* (97%) | - | C+ G+ | Negative for N-acetyl-$\beta$-glucosaminidase, chitin not tested [31] |
| *Halomonas campaniensis* (99%) | ASV3659 (99%) | C+ G+ | No report of GlcNAc or chitin testing [32] |

^1^ Species matches were determined using BLAST searches of the NCBI database.

^2^ ASV matches were determined using a local BLAST search with a database made with all 16S sequences from the first artificial selection experiment.

^3^ Purple indicates that the isolate was able to grow using both chitin (C+) and GlcNAc (G+), red that it could grow using GlcNAc only, and black that it could not grow using either (-).

| 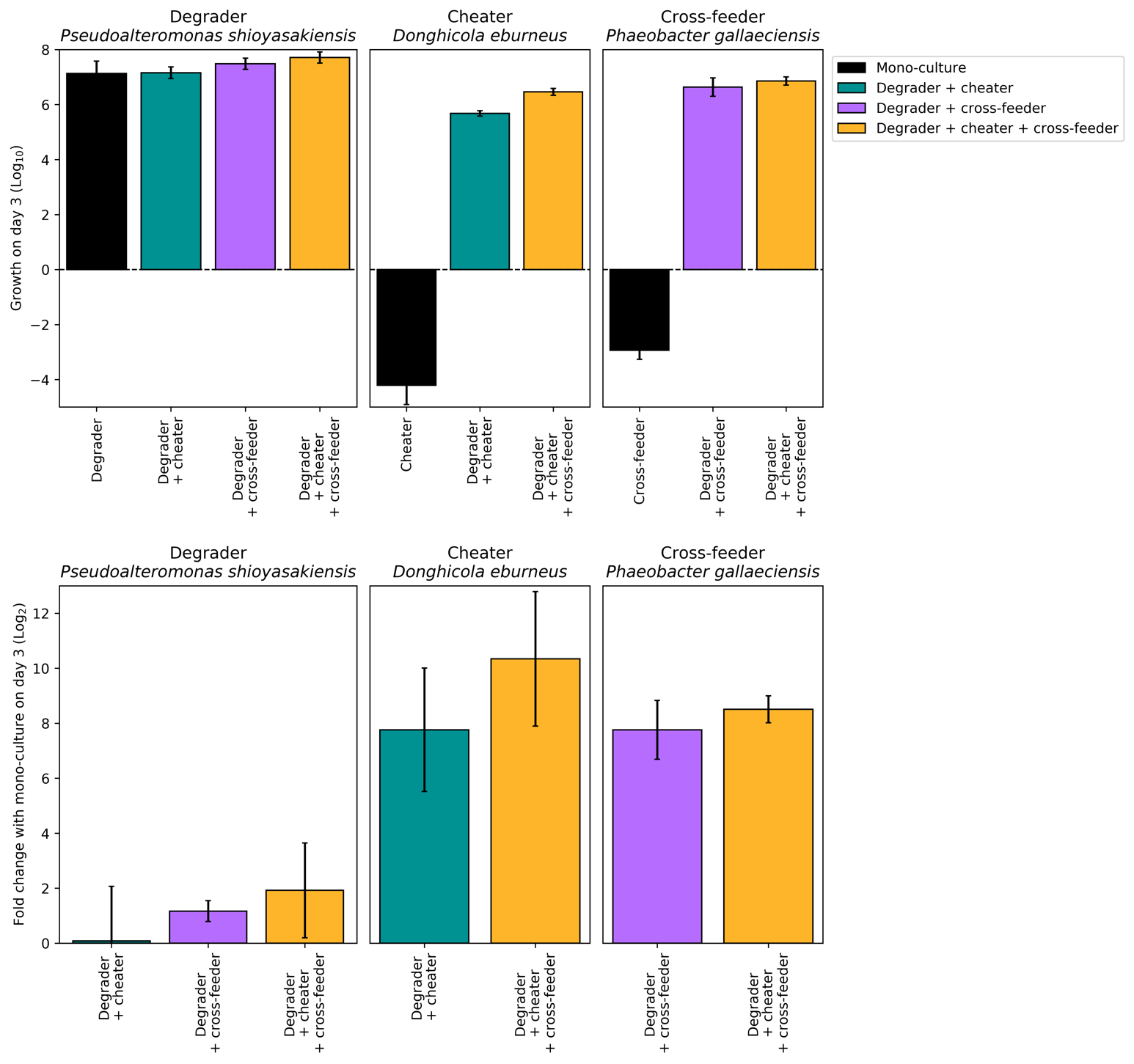 |
| --- |
| **Figure S8**. Growth of a chitin degrader (*Pseudoalteromonas shioyasakaiensis*), a cheater (*i.e.* capable of growth with GlcNAc but not chitin; *Donghicola eburneus*) and a cross-feeder (*i.e.* not capable of growth on either chitin or GlcNAc; *Phaeobacter gallaeciensis*) when grown in mono- and co-cultures. Growth was quantified through qPCR measurements with primers specific to each isolate. Top panels show growth on the third day of incubation (*i.e.* the difference between the measurements on day 0 and day 3) while bottom panels show log_2_ fold change between the mono-cultures and the different co-culture conditions for each isolate (on day 3). Points shown are means of three biological replicates and error bars show standard deviations. |

| 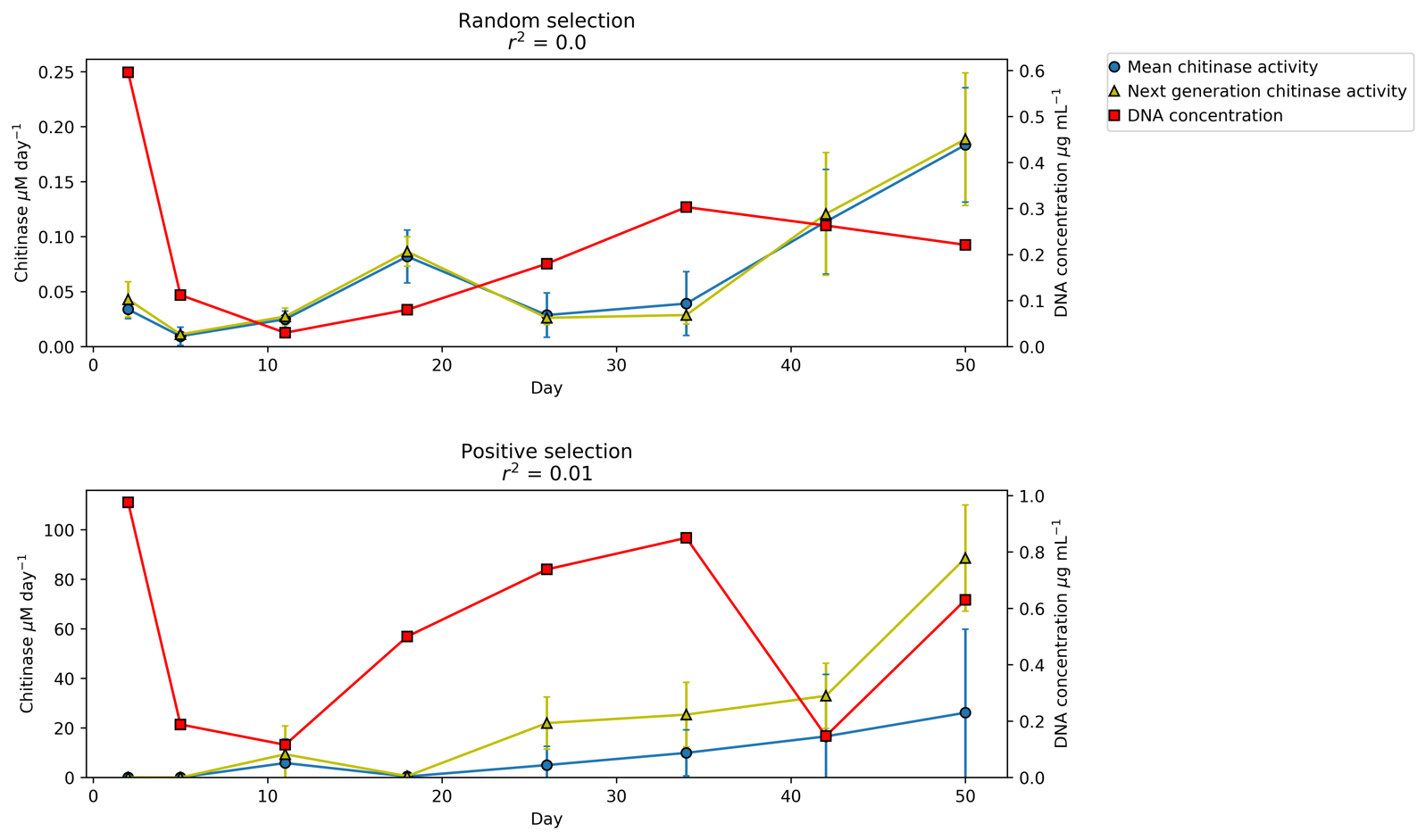 |
| --- |
| **Figure S9**. Averaged absolute chitinase activity measured from all communities (*n*=30; blue lines with circular markers) within each generation, and from the three selected communities used to inoculate the following generation (yellow lines with triangular markers) during artificial selection experiment 2. Red lines with square markers show DNA concentrations for the three communities used to inoculate the next generation and used for 16S rRNA gene amplicon sequencing. Error bars show standard deviations. Note that scales differ between all axis. *r^2^* values indicate Pearson’s correlation coefficients between chitinase activity (next generation) and DNA concentration. |

**References**

1. Hood MA. Comparison of four methods for measuring chitinase activity and the application of the 4-MUF assay in aquatic environments. J Microbiol Methods. 1991;13:151–60.

2. Lecleir GR, Buchan A, Maurer J, Moran MA, Hollibaugh JT. Comparison of chitinolytic enzymes from an alkaline, hypersaline lake and an estuary. Environ Microbiol. 2007;9:197–205.

3. Köllner KE, Carstens D, Keller E, Vazquez F, Schubert CJ, Zeyer J, et al. Bacterial chitin hydrolysis in two lakes with contrasting trophic statuses. Appl Environ Microbiol. 2012;78:695–704.

4. Hall-Stoodley L, Costerton JW, Stoodley P, State M, Engineering B. Bacterial biofilms: from the natural environment to infectious diseases. Nat Rev Microbiol. 2004;2:95–108.

5. Datta MS, Sliwerska E, Gore J, Polz MF, Cordero OX. Microbial interactions lead to rapid micro-scale successions on model marine particles. Nat Commun. 2016;7:1–7.

6. Larimer C, Winder E, Jeters R, Prowant M, Nettleship I, Addleman RS, et al. A method for rapid quantitative assessment of biofilms with biomolecular staining and image analysis. Anal Bioanal Chem. Analytical and Bioanalytical Chemistry; 2016;408:999–1008.

7. Azeredo J, Azevedo NF, Briandet R, Cerca N, Costa AR, Desvaux M, et al. Critical review on biofilm methods. Crit Rev Microbiol. 2017;43:315–51.

8. Mandakhalikar KD, R JN, Chiong E. Extraction and quantification of biofilm bacteria: Method optimized for urinary catheters. Sci Rep. 2018;8:1–9.

9. Guillard RRL, Ryther JH. Studies of Marine Planktonic Diatoms: I. cyclotella, Nana hustedt, and Detonula confervacea (cleve) gran. Can J Microbiol. 1962;8:229–39.

10. Parada AE, Needham DM, Fuhrman JA. Every base matters: assessing small subunit rRNA primers for marine microbiomes with mock communities, time series and global field samples. Environ Microbiol. 2016;18:1403–14.

11. Bradley IM, Pinto AJ, Guest JS. Design and evaluation of Illumina MiSeq-compatible, 18S rRNA gene-specific primers for improved characterization of mixed phototrophic communities. Appl Environ Microbiol. 2016;82:5878–91.

12. Lane DJ. 16S/23S rRNA Sequencing. In: Stackebrandt, E. and Goodfellow M, editor. Nucleic Acid Tech Bact Syst. 1991. p. 115–75.

13. Quan Z-X, Xiao Y-P, Roh SW, Nam Y-D, Chang H-W, Shin K-S, et al. Joostella marina gen. nov., sp. nov., a novel member of the family Flavobacteriaceae isolated from the East Sea. Int J Syst Evol Microbiol. 2008;58:1388–92.

14. Wu YH, Yu PS, Zhou YD, Xu L, Wang CS, Wu M, et al. Muricauda antarctica sp. nov., a marine member of the Flavobacteriaceae isolated from Antarctic seawater. Int J Syst Evol Microbiol. 2013;63:3451–6.

15. Bruns A, Rohde M, Berthe-Corti L. Muricauda ruestringensis gen. nov., sp. nov., a facultatively anaerobic, appendaged bacterium from German North Sea intertidal sediment. Int J Syst Evol Microbiol. 2001;51:1997–2006.

16. Arenskötter M, Bröker D, Steinbüchel A. Biology of the metabolically diverse genus Gordonia. Appl Environ Microbiol. 2004;70:3195–204.

17. Evtushenko LI, Dorofeeva L V., Subbotin SA, Cole JR, Tiedje JM. Leifsonia poae gen. nov., sp. nov., isolated from nematode galls on Poa annua, and reclassification of “Corynebacterium aquaticum” Leifson 1962 as Leifsonia aquatica (ex Leifson 1962) gen. nov., nom. rev., comb. nov. and Clavibacter xyli Davis et al. 1984. Int J Syst Evol Microbiol. 2000;50:371–80.

18. Martens T, Heidorn T, Pukal R, Simon M, Tindall BJ, Brinkhoff T. Reclassification of Roseobacter gallaeciensis Ruiz-Ponte et al. 1998 as Phaeobacter gallaeciensis gen. nov., comb. nov., description of Phaeobacter inhibens sp. nov., reclassification of Ruegeria algicola (Lafay et al. 1995) Uchino et al. 1999 as Marinovu. Int J Syst Evol Microbiol. 2006;56:1293–304.

19. Muramatsu Y, Uchino Y, Kasai H, Suzuki KI, Nakagawa Y. Ruegeria mobilis sp. nov., a member of the Alphaproteobacteria isolated in Japan and Palau. Int J Syst Evol Microbiol. 2007;57:1304–9.

20. Sung HR, Lee JM, Kim M, Yun BR, Shin KS. Donghicola tyrosinivorans sp. Nov., A tyrosinedegrading bacterium isolated from seawater. Int J Syst Evol Microbiol. 2015;65:4140–5.

21. Ramana VV, Kumar PA, Srinivas TNR, Sasikala C, Ramana C V. Rhodobacter aestuarii sp . nov ., a phototrophic alphaproteobacterium isolated from an estuarine environment. Int J Syst Evol Microbiol. 2009;59:1133–6.

22. Wang H, Zhang X, Yan S, Qi Z, Yu Y. Huaishuia halophila gen. nov., sp. nov., isolated from coastal seawater. Int J Syst Evol Microbiol. 2012;62:223–8.

23. Lai Q, Cao J, Yuan J, Li F, Shao Z. Celeribacter indicus sp. nov., a polycyclic aromatic hydrocarbon-degrading bacterium from deep-sea sediment and reclassification of Huaishuia halophila as Celeribacter halophilus comb. nov. Int J Syst Evol Microbiol. 2014;64:4160–7.

24. Zhang R, Lai Q, Wang W, Li S, Shao Z. Thioclava dalianensis sp. nov., isolated from surface seawater. Int J Syst Evol Microbiol. 2013;63:2981–5.

25. Labbé N, Parent S, Villemur R. Nitratireductor aquibiodomus gen. nov., sp. nov., a novel α-proteobacterium from the marine denitrification system of the Montreal Biodome (Canada). Int J Syst Evol Microbiol. 2004;54:269–73.

26. López-Pérez M, Gonzaga A, Martin-Cuadrado AB, Onyshchenko O, Ghavidel A, Ghai R, et al. Genomes of surface isolates of Alteromonas macleodii: The life of a widespread marine opportunistic copiotroph. Sci Rep. 2012;2:1–11.

27. Ivanova EP, Ng HJ, Webb HK, Kurilenko V V., Zhukova N V., Mikhailov V V., et al. Alteromonas australica sp. nov., isolated from the Tasman Sea. Antonie van Leeuwenhoek, Int J Gen Mol Microbiol. 2013;103:877–84.

28. Matsuyama H, Sawazaki K, Minami H, Kasahara H, Horikawa K, Yumoto I. Pseudoalteromonas shioyasakiensis sp . nov ., a marine polysaccharide-producing bacterium. 2017;101–6.

29. Hada HS, West PA, Lee J V., Stemmler J, Colwell RR. Vibrio tubiashii sp. nov., a Pathogen of Bivalve Mollusks. Int J Syst Bacteriol. 1984;34:1–4.

30. Xu XW, Wu YH, Zhou Z, Wang CS, Zhou YG, Zhang H Bin, et al. Halomonas saccharevitans sp. nov., Halomonas arcis sp. nov. and Halomonas subterranea sp. nov., halophilic bacteria isolated from hypersaline environments of China. Int J Syst Evol Microbiol. 2007;57:1619–24.

31. Koh H, Rani S, Kim S, Moon E, Nam SW, Rhee S, et al. Halomonas aestuarii sp . nov ., a moderately halophilic bacterium isolated from a tidal flat. 2018;4298–303.

32. Romano I, Giordano A, Lama L, Nicolaus B, Gambacorta A. Halomonas campaniensis sp. nov., a haloalkaliphilic bacterium isolated from a mineral pool of Campania Region, Italy. Syst Appl Microbiol. 2005;28:610–8.
